# The fusion landscape of hepatocellular carcinoma

**DOI:** 10.1002/1878-0261.12479

**Published:** 2019-04-11

**Authors:** Chengpei Zhu, Liangcai Wu, Yanling Lv, Jinxia Guan, Xue Bai, Jianzhen Lin, Tingting Liu, Xiaobo Yang, Simon C. Robson, Xinting Sang, Chenghai Xue, Haitao Zhao

**Affiliations:** ^1^ Department of Liver Surgery Peking Union Medical College Hospital Chinese Academy of Medical Sciences and Peking Union Medical College Beijing China; ^2^ My Health Gene Technology Co., Ltd. Service Centre of Tianjin Chentang Science and Technology Commercial District China; ^3^ Liver Center and The Transplant Institute Department of Medicine Beth Israel Deaconess Medical Center Harvard Medical School Boston MA USA; ^4^ Joint Laboratory of Large‐scale Medical Data Pattern Mining and Application Institute of Automation Chinese Academy of Sciences Beijing China

**Keywords:** biomarker, gene fusion, hepatocellular carcinoma, recurrent fusion gene

## Abstract

Most cases of hepatocellular carcinoma (HCC) are already advanced at the time of diagnosis, which limits treatment options. Challenges in early‐stage diagnosis may be due to the genetic complexity of HCC. Gene fusion plays a critical function in tumorigenesis and cancer progression in multiple cancers, yet the identities of fusion genes as potential diagnostic markers in HCC have not been investigated. Here, we employed STAR‐Fusion and identified 43 recurrent fusion events in our own and four public RNA‐seq datasets. We identified 2354 different gene fusions in two hepatitis B virus (HBV)‐HCC patients. Validation analysis against the four RNA‐seq datasets revealed that only 1.8% (43/2354) were recurrent fusions. Comparison with the four fusion databases demonstrated that 19 recurrent fusions were not previously annotated to diseases and three were annotated as disease‐related fusion events. Finally, we validated six of the novel fusion events, including RP11‐476K15.1‐CTD‐2015H3.2, by RT‐PCR and Sanger sequencing of 14 pairs of HBV‐related HCC samples. In summary, our study provides new insights into gene fusions in HCC and may contribute to the development of anti‐HCC therapy.

**Abbreviations**
HBV: hepatitis B virus
HCC: hepatocellular carcinoma
NSCLC: non‐small‐cell lung cancer
qRT‐PCR: quantitative real‐time polymerase chain reaction

## 1 Introduction

Hepatocellular carcinoma (HCC) is the third leading cause of cancer‐related death worldwide (Zhou *et al*., 2016). Even though advanced early surveillance technology has improved the life of patients diagnosed at an early stage, most patients are diagnosed with late‐stage of HCC. Furthermore, HCC patients do not show improved long‐term disease‐free survival or overall survival after surgical resection and auxiliary medication (Eggert *et al*., 2013; Kamiyama *et al*., 2009; Llovet *et al*., 2015; Ye *et al*., 2003). One of the main reasons for this may lie in the complexity of the genetic background of HCC (Llovet *et al*., 2015).

Fortunately, recent advances in high throughput sequencing technology have helped provide deeper insights into the genomic and transcriptome landscape of cancer. Using these sequencing technologies, researchers could identify large numbers of mutations, insertions, deletions, and fusions as well as chromosome rearrangements in different types of cancers (Gerlinger *et al*., 2012; Miao *et al*., 2014; Shibata and Aburatani, 2014; Xue *et al*., 2016) . Previous studies have demonstrated that fusion genes play an important role in tumorigenesis and cancer progression (Mitelman *et al*., 2007; Soda *et al*., 2007) and represent one of the most promising therapeutic targets in human malignancy (Cortes *et al*., 2012; Kazandjian *et al*., 2014; Rutkowski *et al*., 2010; Shaw *et al*., 2014). For example, the first fusion gene, Philadelphia chromosome, was discovered in 1960 and approved as the therapeutic biomarker of chronic myeloid leukemia in 2001 (Cohen *et al*., 2002; Nowell, 1960; Topaly *et al*., 2001). In addition, several highly recurrent fusion genes in specific tumor types have been well characterized. For example, Soda *et al*. (2007) showed that nearly 6.7% of non‐small‐cell lung cancer (NSCLC) patients carry the EML4‐ALK fusion. Approximately 55% of prostate cancer showed the presence of ERG fusion (Hessels and Schalken, 2013). The DNAJB1‐PRKACA fusion was found in 100% of fibrolamellar HCC (15/15) (Honeyman *et al*., 2014).

However, the identity of fusion genes in HCC has not been comprehensively investigated. A previous study re‐analyzed RNA‐seq data of normal liver tissue and HepG2 cells from the National Center for Biotechnology Information Sequence Read Archive database and identified 46 fusion genes (Lin *et al*., 2014a). Another study only detected five fusion genes from 11 HCC tissues and 11 paired portal vein tumor thrombus tissues (Zhang *et al*., 2015). Owing to the limited numbers of samples and different analysis strategies, the studies did not identify recurrent fusion genes.

Here we used RNA‐seq data of multiple lesions of two HCC patients to explore gene fusions in HCC and validate potential fusions using publicly available RNA‐seq datasets of HCC. Our efforts unveiled several novel and recurrent fusions in HCC, suggesting their potential as diagnostic markers or molecular therapeutic targets.

## 2 Materials and methods

### 2.1 Patients and clinical samples

**Figure 1:**
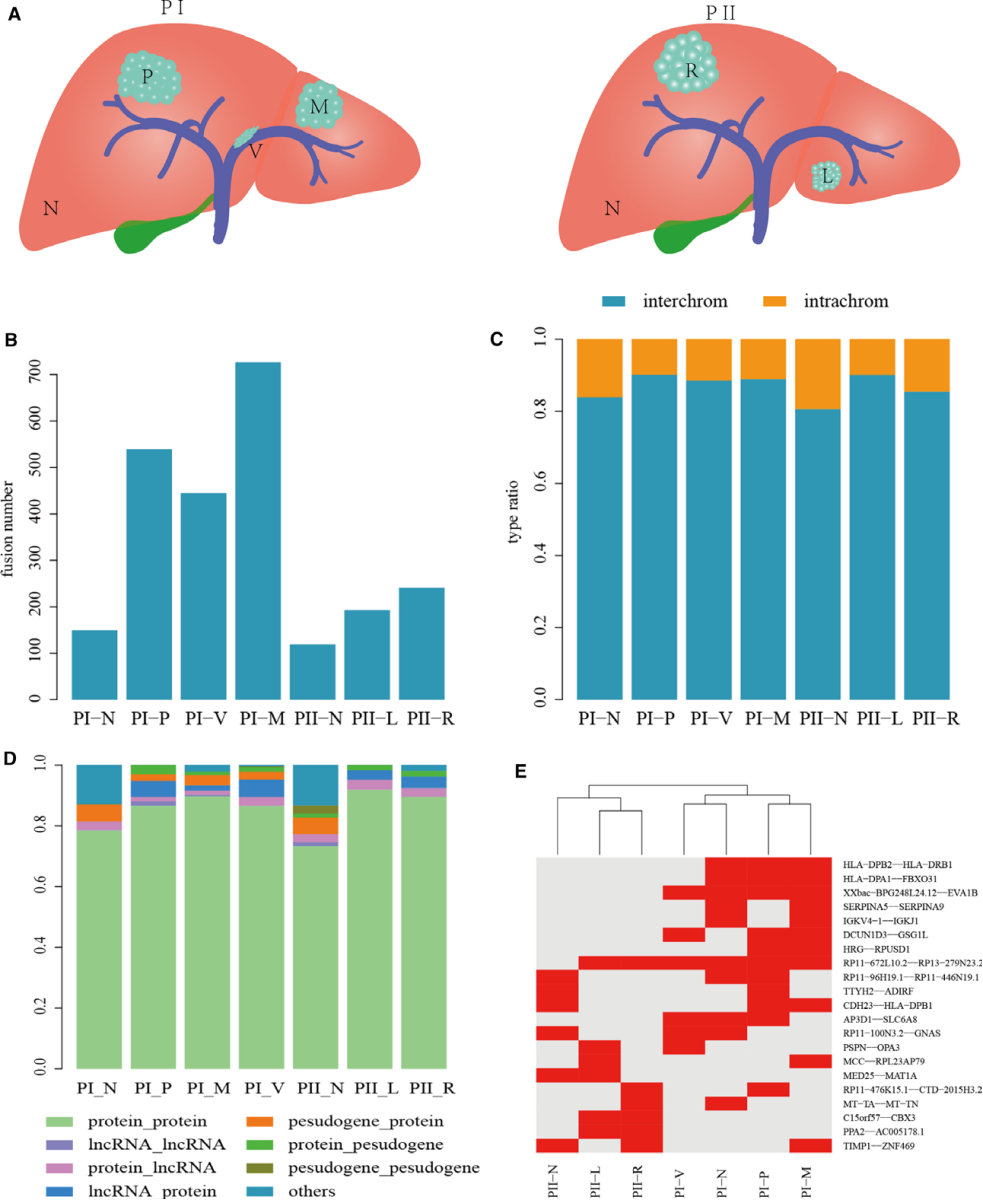
The distribution of fusion events in two multifocal HCC patients*. (A) Ideograph of two multifocal HCC patients. (B) The number of fusion events identified in each sample. (C) The ratio of fusion events between different chromosomes and in the same chromosome. (D) The type of two fusion genes involving fusion events. (E) The clusters of fusions appear at least two samples. *PI, Patient I; PI‐P, primary HCC of Patient I; PI‐M, intrahepatic metastases of Patient I; PI‐V, portal vein tumor thrombus of Patient I; PI‐N, noncancerous liver of Patient I; PII, Patient II; PII‐R, right lobes HCC of Patient II; PII‐L, left lobes HCC of Patient II; PII‐N, noncancerous liver of Patient II.

Two typical multi‐focal hepatitis B virus (HBV)‐HCC patients (Fig. 1A) were enrolled for the study, which was previously reported (Miao *et al*., 2014). The raw RNA‐seq data were deposited at the European Genome‐phenome Archive with accession number EGAS00001000372.

To identify reliable recurrent fusions, we collected publicly available RNA‐seq data of HCC using the keywords ‘hepatocellular carcinoma’, ‘HCC’, ‘liver’, ‘RNA‐seq’, ‘next‐generation sequencing’, and ‘high through output’ in the GEO database and NCBI SRA database. Finally, we obtained four available RNA‐seq datasets [accession numbers: GSE65485 (Dong *et al*., 2015), GSE55758 (Gao *et al*., 2015), GSE33294 (Chan *et al*., 2014) and SRP007560 (Lin *et al*., 2014b)] (Table 1).

**Table 1:** The number of hepatocellular carcinoma and normal liver samples and read length for each public available RNA‐seq data

Accession	Case number	Control number	Read length (bp)
GSE65485	50	5	2 * 100
GSE55758	8	8	2 * 90
SRP007560	1	1	2 * 75
GSE33294	3	3	58

### 2.2 Analysis of transcriptome data

For raw RNA‐seq data, we first assessed the quality of sequencing reads by fastqc software (Babraham Institute, Cambridge, UK) and then discarded low‐quality reads with a quality score < 20 using the trimmomatic tool. Next, for each sample, we aligned the cleaning read to the hg19 reference genome using star v2.4.1 (The Cold Spring Harbor Laboratory, Cold Spring Harbor, NY, USA), an ultrafast RNA‐seq aligner. For each read, no more than two mismatches were allowed in the alignment process. Reads that were mapped to distinct genes in the reference genome were output into the chimeric‐reads file and used for detection of fusion genes.

### 2.3 Identification of fusion transcripts

We used STAR‐Fusion integrated into star software, which was sufficient for fusion RNA prediction compared with other methods (Haas *et al*., 2017; Kumar *et al*., 2016; Nicorici *et al*., 2014; Stransky *et al*., 2014), to identify potential fusion genes. Reads deposited in the chimeric‐reads file indicated putative fusions. STAR‐Fusion used reads that aligned with distinct genes to detect candidate fusion genes. To reduce the number of false‐positive fusion genes, the length of one read aligned to distinct genes was not less than 15 bp. Putative fusions between homologous genes were also discarded. Furthermore, we remained fusions with at least three junction reads, which provided direct evidence about a fusion. We also removed the fusions between mitochondria and autosomes.

### 2.4 Validating of candidate recurrent fusion genes

#### 2.4.1 Patients and clinical samples

Eleven pairs of frozen HBV‐related HCC samples combined with their corresponding adjacent non‐tumor liver tissues and three multiple lesions of patient samples were obtained from HBV‐HCC patients who underwent hepatectomy at Peking Union Medical College Hospital (PUMCH). All patients had pathologically confirmed HCC and did not receive any anticancer treatment prior to surgery. Fresh tissue samples were collected in the operating room and processed within 15 min of resection. Snap‐frozen tissues were stored at −80 °C for subsequent analyses. The experiments were undertaken with the understanding and written consent of each subject. The study methodologies conformed to the standards set by the Declaration of Helsinki and were approved by ethics committee of PUMCH.

#### 2.4.2 RT‐PCR and Sanger sequencing

Total RNA was isolated using TRIzol reagent (Life Technologies, Carlsbad, CA, USA). First‐strand cDNA was synthesized using a High Capacity cDNA Reverse Transcription kit (Life Technologies) according to the manufacturer's instructions. The amplified bands were gel‐purified and later subjected to Sanger sequencing.

#### 2.4.3 Quantitative real‐time PCR (qRT‐PCR)

The cDNA was synthesized from 1.5 μg of total RNA using the High Capacity cDNA Reverse Transcription Kit (Life Technologies). Real‐time PCR was performed using Power SYBR® Green Master mix (Applied Biosystems, Foster City, CA, USA) and a 7500Fast™ Real‐Time PCR System (Applied Biosystems). GAPDH gene expression was included as an internal control. The relative expression levels of the fusion genes were calculated using 2^−ΔΔCT^ values. All statistical analyses were performed using spss 17.0 software (SPSS Inc., Chicago, IL, USA). For statistical comparisons, Student's *t*‐test was performed. The fusion gene‐specific primers and the primers for GAPDH are listed in Table S7.

## 3 Results

### 3.1 Landscape of fusion events in HCC

Transcriptome analysis has been widely used to identify gene fusions in human cancers (Bao *et al*., 2014; Stransky *et al*., 2014; Yoshihara *et al*., 2015). To gain insight into fusion events in HCC, we aligned transcriptome reads to the hg19 reference genome by using star software (Dobin *et al*., 2013). An average of ~ 86 million reads per sample were uniquely mapped to the reference genome (Table 2). We found that 80% (15 302) of expressed genes were protein‐coding genes. We also found that 14% (2651) of expressed genes were long noncoding RNA (lncRNA) and 5% were pseudogenes annotated in GENCODE (Fig. S1). We then used the parameters (Stransky *et al*., 2014) to identify fusion events.

**Table 2:** The library information of RNA‐seq and read mapping for each sample

Sample	Raw read pairs	Clean read pairs	Clean (%)	Unique Map	Unique (%)
PI‐N	43923285	39103125	89.03	37226847	84.75
PI‐P	50352174	44682897	88.74	42769007	84.94
PI‐M	52613930	46523611	88.42	44710681	85
PI‐V	49053998	42656707	86.95	40867848	83.31
PII‐N	45139012	39403503	87.29	37305981	82.65
PII‐L	59886699	51922083	86.7	49206602	82.17
PII‐R	56642919	51940040	91.7	48979659	86.47

As a result, 2354 different gene fusions with more than three junction reads were identified in the seven samples. Among them, HCC samples possessed more fusion events (Fig. 1B) and more involved fusion genes than adjacent normal tissues. Furthermore, the number of gene fusions gradually increased across the PI_N, PI_P, PI_V and PI_M samples (Fig. 1B), which was consistent with intrahepatic metastasis process. The right lesion of patient PII showed 241 fusion events, compared with the 193 of the left lesion. Among the 2354 fusions, we found 20 (0.9%) fusions appearing in more than two samples and they could clearly classify all samples into two patients (Fig. 1E).

We next classified fusion events into eight types according to the gene type. As expected, most fusion events (on average 85%) were between protein‐coding genes in all samples (Fig. 1D). There were fewer fusion types in adjacent normal tissues than those in HCC samples, suggesting that more complex fusions were involved in HCC. Moreover, the proportions of distinct fusion types were different across all samples, and the proportion of protein‐ncRNA fusion events in PI was higher than PII. Further, by analyzing the genome position of fusion genes, we found that more than 85% of fusion events were between two different chromosomes (Fig. 1C) and a few genes fused with more than one partner gene (Fig. S2).

### 3.2 Recurrent fusion events in HCC

**Figure 2:**
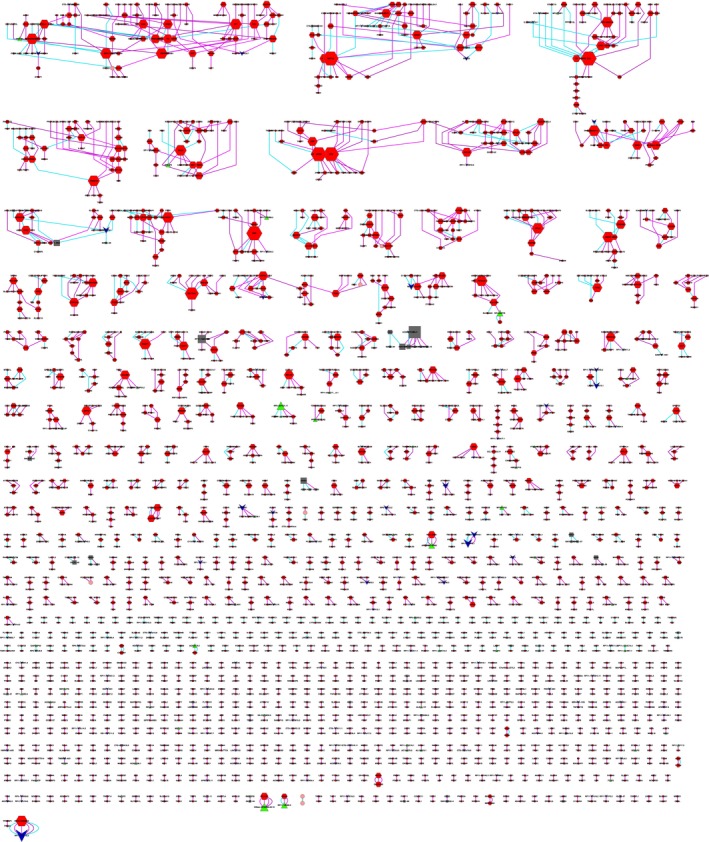
The Fusion Network of two multifocal HCC patients. 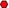 represents protein‐coding genes; 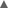 represents pseudogenes; 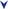 represents lncRNA genes; 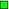 represents other genes; 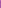 represents fusion event occurred in tumor tissue; 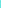 represents fusion event occurred in adjacent non‐tumor tissue.

To obtain recurrent fusions, we conducted the same analysis in four available HCC RNA sequencing datasets. In the public datasets, we obtained 24 960 fusion events in total, including 23 795 fusions in GSE65485, 1132 fusions in GSE55758, 40 fusions in GSE33294, and 92 fusions in SRP007560. In our own data, we obtained 43 gene fusions that occurred in at least two samples in our HCC samples or presented in one sample in our data and also occurred in at least one sample in the 79 public samples (Table S1). The remaining 2311 gene fusions occurred just once in our seven samples, with no events detected in the public data (Fig. 2). Among 2311 gene fusions, we found many kinase genes in fusion events in both HCC and adjacent non‐tumor tissues. For example, MAP3K11 fusion was detected in 23.5% (4/17) of adjacent non‐tumor tissues. Both BRD4 and NRBP2 were detected in 25.8% (16/62) of HCC tissues. MET fusion was only detected in 4.8% (3/62) and 5.9% (1/17) of HCC tissues and adjacent non‐tumor tissues, respectively (Fig. S3).

We speculated whether recurrent gene fusions significantly presented in public samples. We thus grouped the 2354 fusions into two classes: 20 recurrent fusions present in at least two samples in the seven HCC samples, and 2334 fusions that occurred in one of seven samples. We found that 15 recurrent fusions presented in at least one of the 79 public samples and five recurrent fusions only were detected in at least two samples of the seven HCC samples (Table S1). Among the 2334 fusions, 23 fusions were detected in at least one of 79 public samples. We found that recurrent fusions were significantly supported by public datasets (Fisher's exact test, *P*‐value < 2.2e‐16), suggesting that recurrent fusions were likely functional.

### 3.3 Validating candidate recurrent fusion genes in clinical patients with HCC

**Figure 3:**
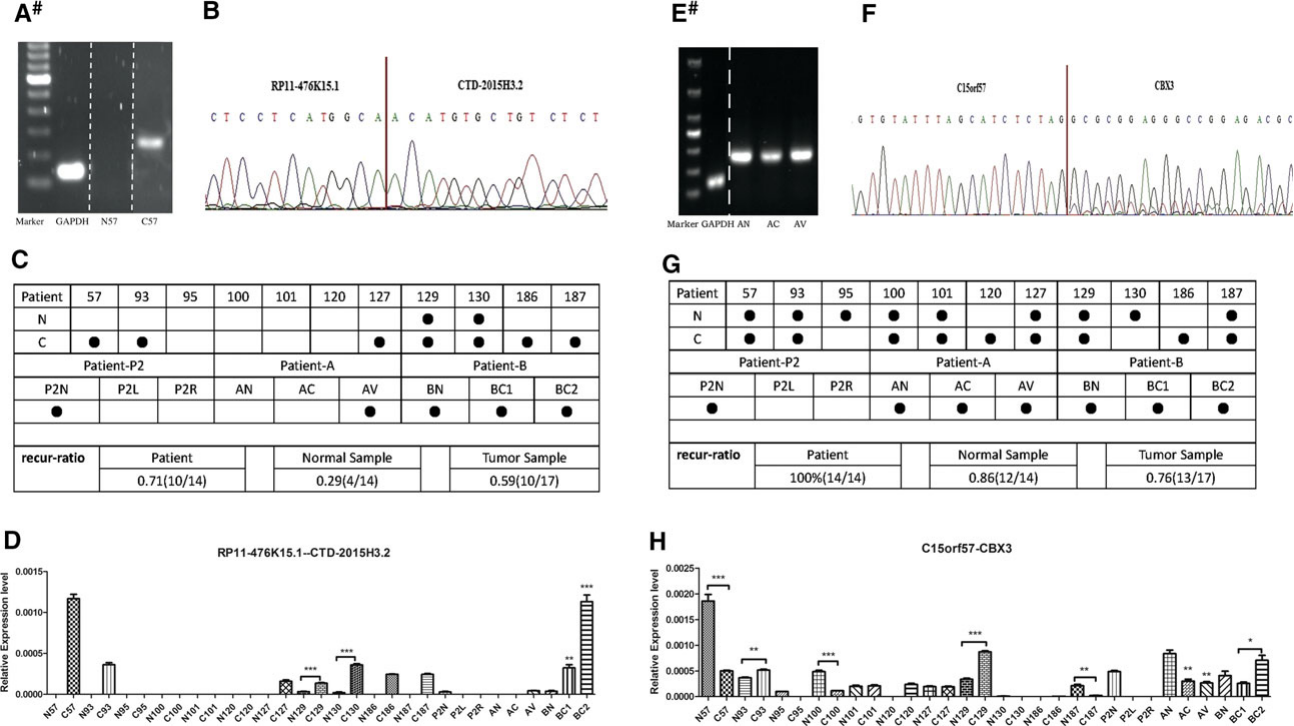
Details of two fusion genes after experimental validation of the fusion transcripts by RT‐PCR, Sanger sequencing and qRT‐PCR. (A,B) The electrophoretic result and sequencing data for RT‐PCR product with fusion gene RP11‐476K15.1–CTD‐2015H3.2. (C) Verified samples for the existence fusion RP11‐476K15.1–CTD‐2015H3.2. Recur‐ratio shows the ratio of the verified sample comparing with the total number in patient, normal (noncancerous) sample and tumor sample, respectively. (D) The relative expression level of fusion gene RP11‐476K15.1–CTD‐2015H3.2 in HCC samples. (E‐H) The result of the fusion gene C15orf57–CBX3; possesses annotation information similar to fusion gene RP11‐476K15.1–CTD‐2015H3.2. ^#^Figures are placed together from different regions of the same gel and separated by a white dotted line. ● represents the verified sample. Data are given as mean ± SEM (*n* = 3). *P < 0.05, **P < 0.01, ***P < 0.001.

We further employed RT‐PCR and Sanger sequencing to validate the 43 recurrent fusion events (Table S6) in 11 pairs of HBV‐related HCC samples combined with their corresponding adjacent non‐tumor liver tissues and three multiple lesion patient samples (Patients II, A and B). Samples from Patient II included noncancerous liver and two distant HCCs located in the left and right lobes; from Patient A, noncancerous liver, tumor lobe and portal vein tumor thrombus; and from patient B, noncancerous liver and two distant HCCs. We successfully obtained primer sequences of 26 fusion genes (Table S7). Six fusion genes were validated to exist in clinical samples (Table 3, Figs 3 and S4–S7). The detailed sequences of these fusion genes are listed in Data S1. Five of the six validated fusion genes (except for IGLV4‐69‐IGLJ3) were detected in many clinical samples in both the tumor samples and adjacent noncancerous samples.

**Table 3:** Details of candidate recurrent fusion genes after experimental validation of the fusion transcripts by RT‐PCR and Sanger sequencing in hepatocellular carcinoma samples

Fusion_name	Chromosome		Supported normal samples	Supported tumor samples
C15orf57–CBX3	Chr15‐chr7	Inter‐	12	13
			N57/N93/N95/N100/N101/N127/N129/N130/N187/P2N/AN/BN	C57/C93/C100/C101/C120/C127/C129/C186/C187/AC/AV/BC1/BC2
IGLV1‐51–IGLL5	Chr22‐chr22	Intra‐	10	13
			N93/N95/N101/N120/N129/N130/N187/P2N/AN/BN	C57/C93/C127/C120/C129/C130/C186/C187/P2L/P2R/AV/BC1/BC2
IGLV4‐69–IGLJ3	Chr22‐chr22	Intra‐	3	2
			N95/N100/P2N	AC/AV
RP11‐100N3.2–GNAS	Chr11‐chr20	Inter‐	9	8
			N57/N93/N95/N100/N101/N127/N129/N186/P2N	C93/C95/C127/C129/C130/C187/P2R/BC1
RP11‐476K15.1–CTD‐2015H3.2	Chr18‐chr18	Intra‐	4	10
			N129/N130/P2N/BN	C57/C93/C127/C129/C130/C186/C187/AV/BC1/BC2
XXbac‐BPG248L24.12–EVA1B	Chr6‐chr1	Inter‐	12	16
			N57/N93/N95/N100/N101/N120/N127/N129/N130/N186/P2N/AN	C57/C93/C95/C101/C120/C127/C129/C130/C186/C187/P2L/P2R/AC/AV/BC1/BC2

We further analyzed the relative gene expression in tumor samples and adjacent noncancerous samples using qRT‐PCR (Figs 3D,H and S4–S7). Among the 26 candidate recurrent fusion genes, six fusions were confirmed by RT‐PCR and Sanger sequencing (Figs 3A,B,E,F and S4–S7). Though some fusions were detected more frequently in tumor samples than in adjacent noncancerous samples, many fusions were frequently detected both in clinical tumor and adjacent noncancerous samples. For instance, the newly identified fusion RP11‐476K15.1‐CTD‐2015H3.2 was detected in our HCC samples PI‐P and PII‐R. For the validated fusions that occurred in tumor and benign samples, relatively higher expression in tumor samples compared with noncancerous samples (Fig. 3A–C). RP11‐476K15.1‐CTD‐2015H3.2 was identified in 71% (10/14) of patients, 29% (4/14) of noncancerous samples and 59% (10/17) of tumor samples (Fig. 3C). These findings suggest that RP11‐476K15.1‐CTD‐2015H3.2 is a novel HCC‐related fusion gene that may be a new therapeutic biomarker or therapy target. Another fusion C15orf57‐CBX3 was detected and showed a considerable expression level in tumor samples and noncancerous samples, similar to another four fusions (Figs 3G,H and S4–S7). C15orf57‐CBX3 was identified in 100% (14/14) of patients, 86% (12/14) of noncancerous samples and 76% (13/17) of tumor samples (Fig. 3G). We suspect that these clinical patients have had a history of HBV infection for several years, taking place in cirrhotic liver, which is not fully normal liver tissue. In the process of liver cirrhosis, the genome of liver tissue changes dramatically, resulting in fusion events.

### 3.4 Identification of candidate fusion events associated with HCC

We further used four known fusion databases including ChiTaRS (Gorohovski *et al*., 2017), ChimerDB (Lee *et al*., 2016), FusionCancer (Wang *et al*., 2015) and Mitelman (Mitelman *et al*., 2016) to examine whether the identified fusion events were associated with human diseases. Three (HLA‐DPB2‐HLA‐DRB1, CDH23‐HLA‐DPB1 and C15orf57‐CBX3) of 43 recurrent gene fusions were annotated as disease‐related fusion events. The fusions HLA‐DPB2‐HLA‐DRB1 and CDH23‐HLA‐DPB1 were both annotated as lung cancer fusion in the FusionCancer database. Additionally, 21 of the remaining 40 recurrent fusion events contained at least one fusion gene that was annotated to participate in at least one disease in the known fusion databases and published literature. For example, IGLV4‐69‐IGLJ3 fusion occurred in PI‐M, and the researcher Sia *et al*. (2015) demonstrated that LOC9610‐IGLJ3 fusion was associated with intrahepatic cholangiocarcinoma. C15orf57‐CBX3, which was present in 18 public HCC samples and four normal liver samples (Table S2, Figs S8 and S10), was also associated with glioblastoma (Bao *et al*., 2014). Moreover, the C15orf57‐CBX3 fusion was associated with cervical cancer, melanoma and Burkitt lymphoma in the ChiTaRS and FusionCancer database. Thus, the C15orf57‐CBX3 fusion may be involved in the development of HCC.

## 4 Discussion

Detection and characterization of fusion genes has been critical in understanding tumorigenesis, anticancer drug screening and clinical application (Hessels and Schalken, 2013; Mertens *et al*., 2015; Stransky *et al*., 2014; Yoshihara *et al*., 2015). However, few fusion events are demonstrated recurrent events. Fortunately, with the development of high throughput sequencing technology as well as bioinformatics algorithms, large amounts of fusion events have been detected (Kim and Salzberg, 2011; Li *et al*., 2013). In the present report, we detected 2354 candidate fusion events and only 1.8% (43/2354) of these events were recurrent. Similarly, Yoshihara *et al*. (2015) and Stransky *et al*. (2014) reported large numbers of kinase gene fusions in 13 and 20 types of cancer, respectively. However, only 7.4 and 12% were recurrent events, respectively.

Several previous studies have focused on fusion events involving protein kinase genes (Stransky *et al*., 2014; Yoshihara *et al*., 2015) because of their critical functions in cellular processes and potential targets for anticancer drugs. In our data analysis, we did not detect protein kinase genes involved in fusion events. This difference may result from the cut‐off value in the detection process. However, some of the identified fusion proteins in our study may have important functionality in HCC. For example, AP3D1‐SLC6A8, which occurred in PI‐N, PI‐P and PI‐V with abundant junction reads, was also present in nine public HCC samples without any supporting normal public liver sample (Table S3, Fig. S10). The AP3D1 gene encodes a protein subunit of the AP3 adaptor‐like complex, which is associated with the Golgi region, as well as more peripheral structures, suggesting an important role in the translation process. The SLC6A8 gene encodes a plasma membrane protein that transports creatine into and out of cells and is also involved in the ion transport process. Furthermore, we found that the breakpoint (chr19:2101455) in the 3′ UTR of AP3D1 does not affect protein domains. The breakpoint (chrX:152961595) of SLC6A8 was also located in the 3′ UTR (Figs S8 and S10). Moreover, AP3D1 was reported to be fused with the NSUN2 gene in HCC in the FusionCancer database. In addition, AP3D1 was reported to be involved in fusions in cervical cancer, lung cancer and colon cancer in the FusionCancer database, indicating that AP3D1 may be widely involved in fusions in human cancer. SLC6A8 was annotated to participate in melanoma (SLC6A10P‐SLC6A8) and glioblastomas (SLC6A8‐GABRA3) in FusionCancer.

In addition to recurrent gene fusions involving known fusion genes or fusion events, we found 19 novel and recurrent fusions that have not been previously annotated to diseases (Table S6). For instance, the recurrent fusion DCUN1D3‐GSG1L occurred in PI‐P, PI‐V and PI‐M without any public sample support (Table S4). Overexpressing DCUN1D3 gene may promote mesenchymal to epithelial‐like changes and inhibit colony formation in soft agar (Huang *et al*., 2014). GSG1L is a component of the inner core of the AMPAR complex, which modifies AMPA receptor gating. Furthermore, both fusion genes harbored the exact same breakpoint in three HCC samples (Fig. S9, Table S4). The breakpoint (chr16:20871370) in the third exon of DCUN1D3 is located in the DUF298‐conserved domain that binds to cullins and Rbx‐1, components of an E3 ubiquitin ligase complex for neddylation. The protein structure is affected by the fusion. The breakpoint in the sixth exon of GSG1L (chr16:27802788) is located downstream of the protein's conserved domain without affecting any domains. Another novel recurrent fusion SERPINA5‐SERPINA9 occurred in PI‐N and PI‐M, as well as in nine public HCC samples (Table S5, Fig. S9). SERPINA5 is a serpin peptidase inhibitor with serine‐type endopeptidase inhibitor activity. The breakpoint (chr14:95053889) in SERPINA5 and the breakpoint (chr14:94935978) in SERPINA9 were both located in conserved domains of each protein, suggesting that the domains are truncated in the fusion protein. The fusion can generate a chimeric protein that may be involved in the tumorigenesis of HCC and should be further validated. Thus, DCUN1D3‐GSG1L and SERPINA5‐SERPINA9 may be involved in the development of HCC and should be examined.

Noncoding genes also play an important role in human disorders, human pluripotency and cancers (Guarnerio *et al*., 2016; Xu *et al*., 2017; Yu *et al*., 2018). However, to our best knowledge, up until now, few studies have focused on noncoding gene fusion events. Lau *et al*. (2014) reported that HBx‐LINE fusion, which functions as an lncRNA, affected β‐catenin transitivity and was involved in liver cancer development and progression. Qin *et al*. (2016,2017) showed that SLC45A3‐ELK4 and D2HGDH‐GAL3ST2 regulate cancer cell proliferation and cell motility in prostate cancer. Dong *et al*. (2015) found that HCC patients carrying the HBV‐MLL4 fusion have a distinct gene expression profile. In our analysis, we identified many fusion events involving noncoding genes. Notably, a higher percentage of noncoding gene fusion events were detected in the advanced HCC patient. This supports the idea that noncoding gene fusions play key roles in the progression of cancer.

We also detected many recurrent fusion events in adjacent normal tissue. For example, 27.9% (12/43) of fusion events were detected in only adjacent normal tissues and 30.2% (13/43) recurrent fusion events were observed in both HCC and adjacent normal tissues. Some were highly detected in adjacent normal tissue compared with the paired HCC tissue. Similarly, the TEL‐AML1 fusion gene was reported to occur 100 times more frequently in normal individuals than in leukemia patients, and contributes to initiation of childhood ALL (Mori *et al*., 2002; Zelent *et al*., 2004). Thus, fusion events in adjacent normal tissue may serve as biomarkers of hepatitis disease progression into HCC and should be pursued in future experimental studies. Successfully applied drugs targeting BCR‐ABL1 fusion in hematological malignancy ALK fusion in NSCLC, have dramatically ignited enthusiasm for deep exploration of the landscape of gene fusions (Mertens *et al*., 2015). Moreover, several drugs, such as imatinib and crizotinib, have been approved for targeting gene fusions in human malignant diseases. These success stories support the notion that fusion events represent promising anticancer targets. Our present result provides insight into the landscape of gene fusions in HCC and might pave the way for anti‐HCC therapy.

## 5 Conclusions

In our study, we conducted analysis of RNA‐seq data of 67 HCC tissues and 19 adjacent normal tissues to describe the fusion landscape of HCC. As a result, we identified 27 314 non‐redundant fusion events. Among them, 43 recurrent fusions were identified. Except for protein‐protein gene fusion, in our analysis we also found that a lot of noncoding sequences could participate in gene fusion. Finally, we validated six of the novel fusion events by RT‐PCR and Sanger sequencing. Our study provides new insights into gene fusions in HCC and could contribute to the development of anti‐HCC therapy. These findings may broaden our horizon about fusion events in HCC.

## Conflict of interest

The authors declare no conflict of interest.

## Author contributions

SCR, XTS, CHX and HHZ designed the study. YLL, JXG and TTL acquired, analyzed and interpreted the data. CPZ, LCW, XB, JZL and XBY completed the experimental part. CPZ, LCW, YLL and JXG wrote the manuscript. Funding was obtained by XTS and HTZ. All authors critically revised the manuscript and approved the final manuscript.

## Supporting information


**Fig. S1.** (A) The transcriptome component of liver tissue the classification of lncRNAs. (B,C) The patient‐specific transcriptome.

**Fig. S2.** The number of partner genes of each fusion genes in each samples.

**Fig. S3.** Kinase gene fusions in HCC.

**Fig. S4.** Details of IGLV1‐51–IGLL5 after experimental validation of the fusion transcripts by RT‐PCR, Sanger sequencing and qRT‐PCR.

**Fig. S5.** Details of RP11‐100N3.2–GNAS after experimental validation of the fusion transcripts by RT‐PCR, Sanger sequencing and qRT‐PCR.

**Fig. S6.** Details of XXbac‐BPG248L24.12–EVA1B after experimental validation of the fusion transcripts by RT‐PCR, Sanger sequencing and qRT‐PCR.

**Fig. S7.** Details of IGLV4‐69–IGLJ3 after experimental validation of the fusion transcripts by RT‐PCR and Sanger sequencing.

**Fig. S8.**The fusion events involved in known disease related fusion genes. (A) The breakpoint of known disease fusion C15orf57–CBX3. (B) The breakpoint of AP3D1—SLC6A8.

**Fig. S9.**The breakpoint of novel fusion events associated with HCC. (A) The breakpoint of DCUN1D3–GSG1L. (B) The breakpoint of SERPINA5–SERPINA9.

**Fig. S10.** The junction reads of two known disease‐related fusion genes. (A) The junction reads of C15orf57‐CBX3. (B) The junction reads of AP3D1‐SLC6A8.

**Table S1.** The number of fusions with fusions occurring once and recurrent fusions supported by public HCC samples.

**Table S2.** The breakpoint and junction reads of C15orf57–CBX3 across all samples where it occurred.

**Table S3.** The breakpoint and junction reads of AP3D1–SLC6A8 across all samples where it occurred.

**Table S4.** The breakpoint and junction reads of DCUN1D3–GSG1L across all samples where it occurred.

**Table S5.** The breakpoint and junction reads of SERPINA5–SERPINA9 across all samples where is occurred.

**Table S6.** The detail information of 43 candidate recurrent fusion genes.

**Table S7.** The primer sequences for candidate recurrent fusion genes and internal control gene(GAPDH).

**Data S1.** The sequences for validation recurrent fusion genes.

## References

[mol212479-bib-0001] Bao ZS , Chen HM , Yang MY , Zhang CB , Yu K , Ye WL , Hu BQ , Yan W , Zhang W , Akers J *et al* (2014) RNA‐seq of 272 gliomas revealed a novel, recurrent PTPRZ1‐MET fusion transcript in secondary glioblastomas. Genome Res 24, 1765–1773. 25135958 10.1101/gr.165126.113 PMC4216918

[mol212479-bib-0002] Chan TH , Lin CH , Qi L , Fei J , Li Y , Yong KJ , Liu M , Song Y , Chow RK , Ng VH *et al* (2014) A disrupted RNA editing balance mediated by ADARs (Adenosine DeAminases that act on RNA) in human hepatocellular carcinoma. Gut 63, 832–843. 23766440 10.1136/gutjnl-2012-304037 PMC3995272

[mol212479-bib-0003] Cohen MH , Williams G , Johnson JR , Duan J , Gobburu J , Rahman A , Benson K , Leighton J , Kim SK , Wood R *et al* (2002) Approval summary for imatinib mesylate capsules in the treatment of chronic myelogenous leukemia. Clin Cancer Res 8, 935–942. 12006504

[mol212479-bib-0004] Cortes JE , Kim DW , Kantarjian HM , Brummendorf TH , Dyagil I , Griskevicius L , Malhotra H , Powell C , Gogat K , Countouriotis AM *et al* (2012) Bosutinib versus imatinib in newly diagnosed chronic‐phase chronic myeloid leukemia: results from the BELA trial. J Clin Oncol 30, 3486–3492. 22949154 10.1200/JCO.2011.38.7522 PMC4979199

[mol212479-bib-0005] Dobin A , Davis CA , Schlesinger F , Drenkow J , Zaleski C , Jha S , Batut P , Chaisson M and Gingeras TR (2013) STAR: ultrafast universal RNA‐seq aligner. Bioinformatics 29, 15–21. 23104886 10.1093/bioinformatics/bts635 PMC3530905

[mol212479-bib-0006] Dong H , Zhang L , Qian Z , Zhu X , Zhu G , Chen Y , Xie X , Ye Q , Zang J , Ren Z *et al* (2015) Identification of HBV‐MLL4 integration and its molecular basis in Chinese hepatocellular carcinoma. PLoS ONE 10, e0123175. 25901726 10.1371/journal.pone.0123175 PMC4406717

[mol212479-bib-0007] Eggert T , McGlynn KA , Duffy A , Manns MP , Greten TF and Altekruse SF (2013) Epidemiology of fibrolamellar hepatocellular carcinoma in the USA, 2000‐10. Gut 62, 1667–1668. 23708586 10.1136/gutjnl-2013-305164 PMC4145809

[mol212479-bib-0008] Gao F , Liang H , Lu H , Wang J , Xia M , Yuan Z , Yao Y , Wang T , Tan X , Laurence A *et al* (2015) Global analysis of DNA methylation in hepatocellular carcinoma by a liquid hybridization capture‐based bisulfite sequencing approach. Clin Epigenet 7, 86. 10.1186/s13148-015-0121-1 PMC4546208 26300991

[mol212479-bib-0009] Gerlinger M , Rowan AJ , Horswell S , Larkin J , Endesfelder D , Gronroos E , Martinez P , Matthews N , Stewart A , Tarpey P *et al* (2012) Intratumor heterogeneity and branched evolution revealed by multiregion sequencing. N Engl J Med 366, 883–892. 22397650 10.1056/NEJMoa1113205 PMC4878653

[mol212479-bib-0010] Gorohovski A , Tagore S , Palande V , Malka A , Dorith RS and Milana FM (2017) ChiTaRS‐3.1‐the enhanced chimeric transcripts and RNA‐seq database matched with protein‐protein interactions. Nucleic Acids Res 45, D790–D795. 27899596 10.1093/nar/gkw1127 PMC5210585

[mol212479-bib-0011] Guarnerio J , Bezzi M , Jeong JC , Paffenholz SV , Berry K , Naldini MM , Lo‐Coco F , Tay Y , Beck AH and Pandolfi PP . (2016). Oncogenic role of fusion‐circRNAs derived from cancer‐associated chromosomal translocations. Cell 165, 289–302. 27040497 10.1016/j.cell.2016.03.020

[mol212479-bib-0012] Haas B , Dobin A , Stransky N , Li B , Yang X , Tickle T , Bankapur A , Ganote C , Doak T , Pochet N *et al* (2017). STAR‐fusion: fast and accurate fusion transcript detection from RNA‐Seq. bioRxiv120295; 10.1101/120295 [PREPRINT].

[mol212479-bib-0013] Hessels D and Schalken JA (2013) Recurrent gene fusions in prostate cancer: their clinical implications and uses. Curr Urol Rep 14, 214–222. 23625457 10.1007/s11934-013-0321-1

[mol212479-bib-0014] Honeyman JN , Simon EP , Robine N , Chiaroni‐Clarke R , Darcy DG , Lim II , Gleason CE , Murphy JM , Rosenberg BR , Teegan L *et al* (2014) Detection of a recurrent DNAJB1‐PRKACA chimeric transcript in fibrolamellar hepatocellular carcinoma. Science 343, 1010–1014. 24578576 10.1126/science.1249484 PMC4286414

[mol212479-bib-0015] Huang G , Stock C , Bommelje CC , Weeda VB , Shah K , Bains S , Buss E , Shaha M , Rechler W , Ramanathan SY *et al* (2014) SCCRO3 (DCUN1D3) antagonizes the neddylation and oncogenic activity of SCCRO (DCUN1D1). J Biol Chem 289, 34728–34742. 25349211 10.1074/jbc.M114.585505 PMC4263876

[mol212479-bib-0016] Kamiyama T , Nakanishi K , Yokoo H , Kamachi H , Tahara M , Suzuki T , Shimamura T , Furukawa H , Matsushita M and Todo S (2009) Recurrence patterns after hepatectomy of hepatocellular carcinoma: implication of Milan criteria utilization. Ann Surg Oncol 16, 1560–1571. 19259739 10.1245/s10434-009-0407-7

[mol212479-bib-0017] Kazandjian D , Blumenthal GM , Chen HY , He K , Patel M , Justice R , Keegan P and Pazdur R (2014) FDA approval summary: crizotinib for the treatment of metastatic non‐small cell lung cancer with anaplastic lymphoma kinase rearrangements. Oncologist 19, e5–e11. 25170012 10.1634/theoncologist.2014-0241 PMC4201002

[mol212479-bib-0018] Kim D and Salzberg SL (2011) TopHat‐Fusion: an algorithm for discovery of novel fusion transcripts. Genome Biol 12, R72. 21835007 10.1186/gb-2011-12-8-r72 PMC3245612

[mol212479-bib-0019] Kumar S , Vo AD , Qin F and Li H (2016) Comparative assessment of methods for the fusion transcripts detection from RNA‐Seq data. Sci Rep 6, 21597. 26862001 10.1038/srep21597 PMC4748267

[mol212479-bib-0020] Lau CC , Sun T , Ching AK , He M , Li JW , Wong AM , Co NN , Chan AW , Li PS , Lung RW *et al* (2014) Viral‐human chimeric transcript predisposes risk to liver cancer development and progression. Cancer Cell 25, 335–349. 24582836 10.1016/j.ccr.2014.01.030

[mol212479-bib-0021] Lee M , Lee K , Yu N , Jang IN , Choi IK , Kim P , Jang YE , Kim B , Kim S , Lee B *et al* (2016). ChimerDB 3.0: an enhanced database for fusion genes from cancer transcriptome and literature data mining. Nucleic Acids Res 45: D784–D789. 27899563 10.1093/nar/gkw1083 PMC5210563

[mol212479-bib-0022] Li JW , Wan R , Yu CS , Co NN , Wong N and Chan TF (2013) ViralFusionSeq: accurately discover viral integration events and reconstruct fusion transcripts at single‐base resolution. Bioinformatics 29, 649–651. 23314323 10.1093/bioinformatics/btt011 PMC3582262

[mol212479-bib-0023] Lin J , Song X and Liu C (2014a) Pelvic intravascular leiomyomatosis associated with benign pulmonary metastasizing leiomyoma: clinicopathologic, clonality, and copy number variance analysis. Int J Gynecol Pathol 33, 140–145. 24487468 10.1097/PGP.0b013e31828def26

[mol212479-bib-0024] Lin KT , Shann YJ , Chau GY , Hsu CN and Huang CY (2014b) Identification of latent biomarkers in hepatocellular carcinoma by ultra‐deep whole‐transcriptome sequencing. Oncogene 33, 4786–4794. 24141781 10.1038/onc.2013.424

[mol212479-bib-0025] Llovet JM , Villanueva A , Lachenmayer A and Finn RS (2015) Advances in targeted therapies for hepatocellular carcinoma in the genomic era. Nat Rev Clin Oncol 12, 408–424. 26054909 10.1038/nrclinonc.2015.103

[mol212479-bib-0026] Mertens F , Johansson B , Fioretos T and Mitelman F (2015) The emerging complexity of gene fusions in cancer. Nat Rev Cancer 15, 371–381. 25998716 10.1038/nrc3947

[mol212479-bib-0027] Miao R , Luo H , Zhou H , Li G , Bu D , Yang X , Zhao X , Zhang H , Liu S , Zhong Y *et al* (2014) Identification of prognostic biomarkers in hepatitis B virus‐related hepatocellular carcinoma and stratification by integrative multi‐omics analysis. J Hepatol 61, 840–849. 24859455 10.1016/j.jhep.2014.05.025

[mol212479-bib-0028] Mitelman F , Johansson B and Mertens F (2007) The impact of translocations and gene fusions on cancer causation. Nat Rev Cancer 7, 233–245. 17361217 10.1038/nrc2091

[mol212479-bib-0029] Mitelman F , Johansson B and Mertens F (2016). Mitelman Database of Chromosome Aberrations and Gene Fusions in Cancer.

[mol212479-bib-0030] Mori H , Colman SM , Xiao Z , Ford AM , Healy LE , Donaldson C , Hows JM , Navarrete C and Greaves M (2002) Chromosome translocations and covert leukemic clones are generated during normal fetal development. Proc Natl Acad Sci U S A 99, 8242–8247. 12048236 10.1073/pnas.112218799 PMC123052

[mol212479-bib-0031] Nicorici D , Satalan M , Edgren H , Kangaspeska S , Murumagi A , Kallioniemi O , Virtanen S and Kilkku O (2014). FusionCatcher – a tool for finding somatic fusion genes in paired‐end RNA‐sequencing data. bioRxiv. [PREPRINT].

[mol212479-bib-0032] Nowell P (1960) A minute chromosome in human chronic granulocytic leukemia. Science 132, 1497.

[mol212479-bib-0033] Qin F , Song Z , Chang M , Song Y , Frierson H and Li H (2016) Recurrent cis‐SAGe chimeric RNA, D2HGDH‐GAL3ST2, in prostate cancer. Cancer Lett 28, 39–46. 10.1016/j.canlet.2016.06.013 PMC5553067 27322736

[mol212479-bib-0034] Qin F , Zhang Y , Liu J and Li H (2017) SLC45A3‐ELK4 functions as a long non‐coding chimeric RNA. Cancer Lett 28, 53–61. 10.1016/j.canlet.2017.07.007 28716526

[mol212479-bib-0035] Rutkowski P , Van Glabbeke M , Rankin CJ , Ruka W , Rubin BP , Debiec‐Rychter M , Lazar A , Gelderblom H , Sciot R , Lopez‐Terrada D *et al* (2010) Imatinib mesylate in advanced dermatofibrosarcoma protuberans: pooled analysis of two phase II clinical trials. J Clin Oncol 28, 1772–1779. 20194851 10.1200/JCO.2009.25.7899 PMC3040044

[mol212479-bib-0036] Shaw AT , Kim DW , Mehra R , Tan DS , Felip E , Chow LQ , Camidge DR , Vansteenkiste J , Sharma S , De Pas T *et al* (2014) Ceritinib in ALK‐rearranged non‐small‐cell lung cancer. N Engl J Med 370, 1189–1197. 24670165 10.1056/NEJMoa1311107 PMC4079055

[mol212479-bib-0037] Shibata T and Aburatani H (2014) Exploration of liver cancer genomes. Nat Rev Gastroenterol Hepatol 11, 340–349. 24473361 10.1038/nrgastro.2014.6

[mol212479-bib-0038] Sia D , Losic B , Moeini A , Cabellos L , Hao K , Revill K , Bonal D , Miltiadous O , Zhang Z , Hoshida Y *et al* (2015) Massive parallel sequencing uncovers actionable FGFR2‐PPHLN1 fusion and ARAF mutations in intrahepatic cholangiocarcinoma. Nat Commun 6, 6087. 25608663 10.1038/ncomms7087

[mol212479-bib-0039] Soda M , Choi YL , Enomoto M , Takada S , Yamashita Y , Ishikawa S , Fujiwara S , Watanabe H , Kurashina K , Hatanaka H *et al* (2007) Identification of the transforming EML4‐ALK fusion gene in non‐small‐cell lung cancer. Nature 448, 561–566. 17625570 10.1038/nature05945

[mol212479-bib-0040] Stransky N , Cerami E , Schalm S , Kim JL and Lengauer C (2014) The landscape of kinase fusions in cancer. Nat Commun 5, 4846. 25204415 10.1038/ncomms5846 PMC4175590

[mol212479-bib-0041] Topaly J , Zeller WJ and Fruehauf S (2001) Synergistic activity of the new ABL‐specific tyrosine kinase inhibitor STI571 and chemotherapeutic drugs on BCR‐ABL‐positive chronic myelogenous leukemia cells. Leukemia 15, 342–347. 11237055 10.1038/sj.leu.2402041

[mol212479-bib-0042] Wang Y , Wu N , Liu J , Wu Z and Dong D (2015) FusionCancer: a database of cancer fusion genes derived from RNA‐seq data. Diagn Pathol 10, 131. 26215638 10.1186/s13000-015-0310-4 PMC4517624

[mol212479-bib-0043] Xu J , Bai J , Zhang X , Lv Y , Gong Y , Liu L , Zhao H , Yu F , Ping Y , Zhang G *et al* (2017) A comprehensive overview of lncRNA annotation resources. Brief Bioinform 18, 236–249. 26944085 10.1093/bib/bbw015

[mol212479-bib-0044] Xue R , Li R , Guo H , Guo L , Su Z , Ni X , Qi L , Zhang T , Li Q , Zhang Z *et al* (2016) Variable Intra‐tumor genomic heterogeneity of multiple lesions in patients with hepatocellular carcinoma. Gastroenterology 150, 998–1008. 26752112 10.1053/j.gastro.2015.12.033

[mol212479-bib-0045] Ye QH , Qin LX , Forgues M , He P , Kim JW , Peng AC , Simon R , Li Y , Robles AI , Chen Y *et al* (2003) Predicting hepatitis B virus‐positive metastatic hepatocellular carcinomas using gene expression profiling and supervised machine learning. Nat Med 9, 416–423. 12640447 10.1038/nm843

[mol212479-bib-0046] Yoshihara K , Wang Q , Torres‐Garcia W , Zheng S , Vegesna R , Kim H and Verhaak RG (2015) The landscape and therapeutic relevance of cancer‐associated transcript fusions. Oncogene 34, 4845–4854. 25500544 10.1038/onc.2014.406 PMC4468049

[mol212479-bib-0047] Yu CY , Chuang CY and Kuo HC (2018) Trans‐spliced long non‐coding RNA: an emerging regulator of pluripotency. Cell Mol Life Sci 75, 3339–3351. 29961157 10.1007/s00018-018-2862-4 PMC11105688

[mol212479-bib-0048] Zelent A , Greaves M and Enver T (2004) Role of the TEL‐AML1 fusion gene in the molecular pathogenesis of childhood acute lymphoblastic leukaemia. Oncogene 23, 4275–4283. 15156184 10.1038/sj.onc.1207672

[mol212479-bib-0049] Zhang H , Ye J , Weng X , Liu F , He L , Zhou D and Liu Y (2015) Comparative transcriptome analysis reveals that the extracellular matrix receptor interaction contributes to the venous metastases of hepatocellular carcinoma. Cancer Genet 208, 482–491. 26271415 10.1016/j.cancergen.2015.06.002

[mol212479-bib-0050] Zhou M , Wang H , Zhu J , Chen W , Wang L , Liu S , Li Y , Wang L , Liu Y , Yin P *et al* (2016) Cause‐specific mortality for 240 causes in China during 1990‐2013: a systematic subnational analysis for the Global Burden of Disease Study 2013. Lancet 387, 251–272. 26510778 10.1016/S0140-6736(15)00551-6

