# Supplementary figures and images for: The fusion landscape of hepatocellular carcinoma
Source: Mol Oncol. 2019 Apr 11;13(5):1214–25. doi: 10.1002/1878-0261.12479 (PMC6487730; doi:10.1002/1878-0261.12479)

A

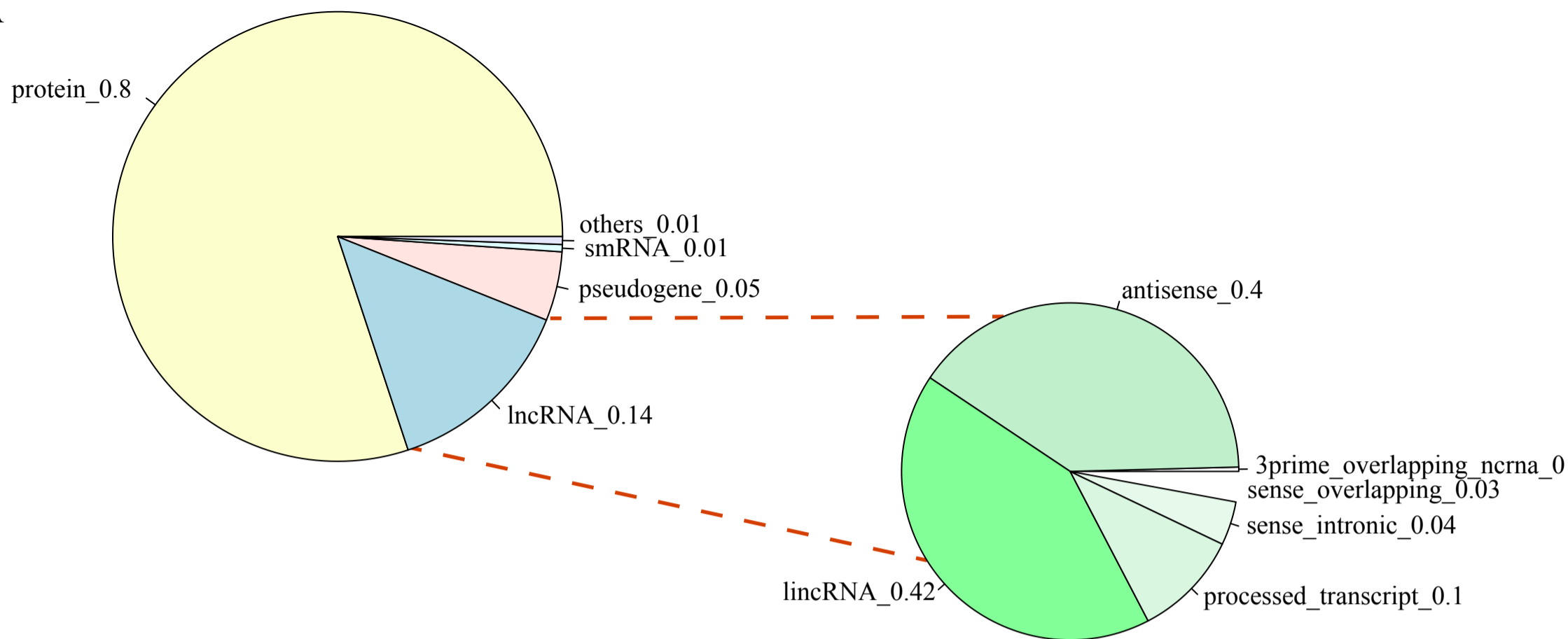

B

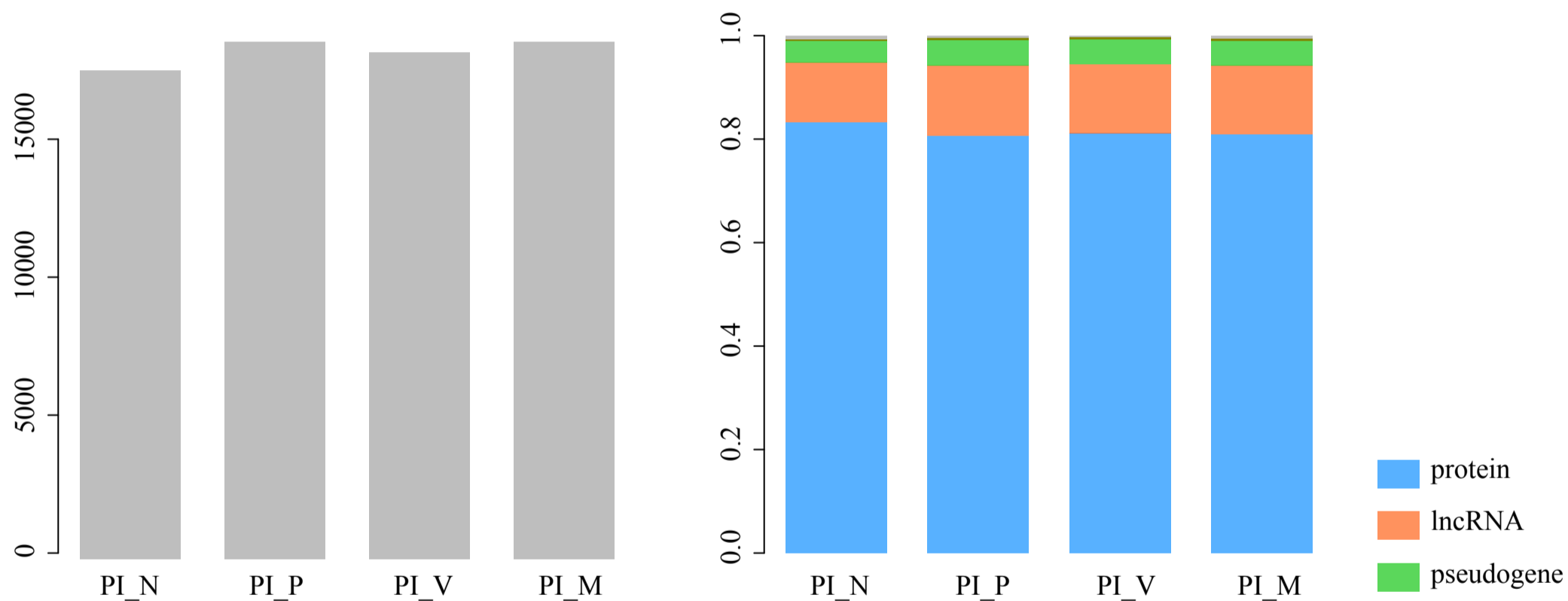

C

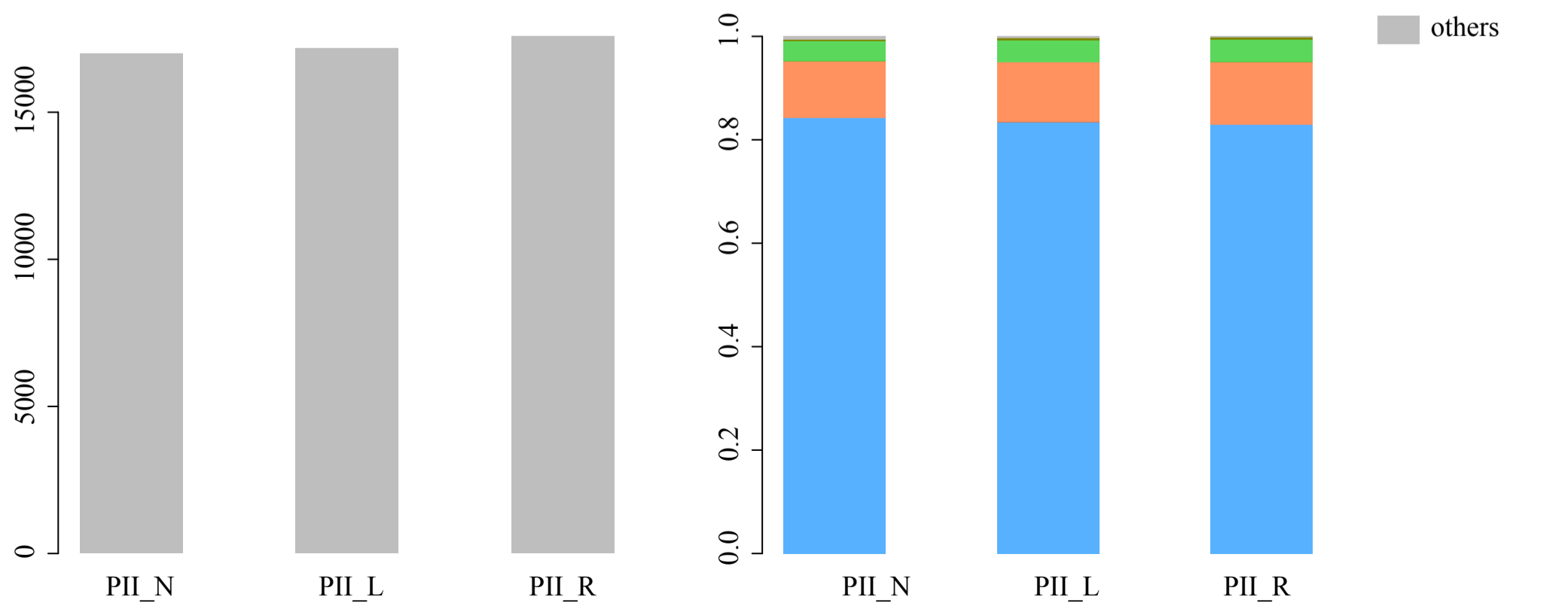

Supplement: Supplementary file 1 — Fig. S1. (A) The transcriptome component of liver tissue the classification of lncRNAs. (B,C) The patient‐specific transcriptome. [file MOL2-13-1214-s001.pdf]

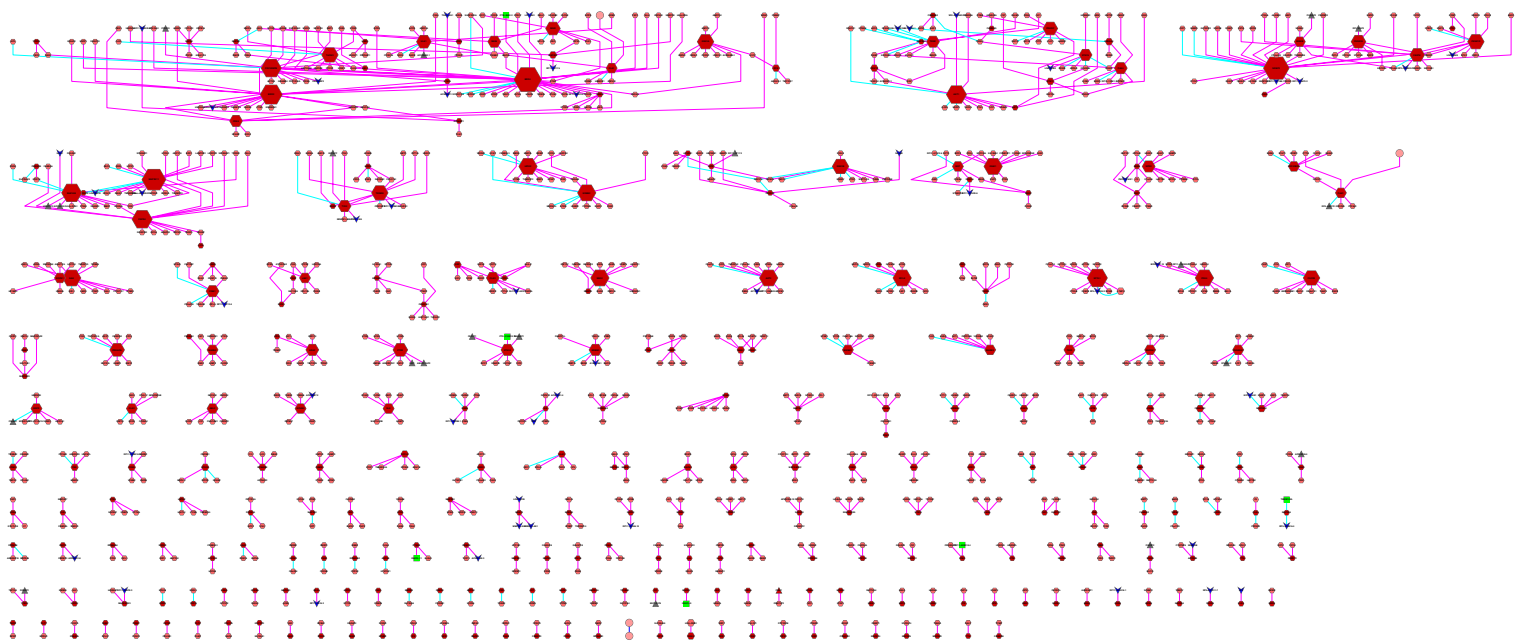

Supplement: Supplementary file 3 — Fig. S3. Kinase gene fusions in HCC. [file MOL2-13-1214-s003.pdf]

A

C15orf57--CBX3 junction read

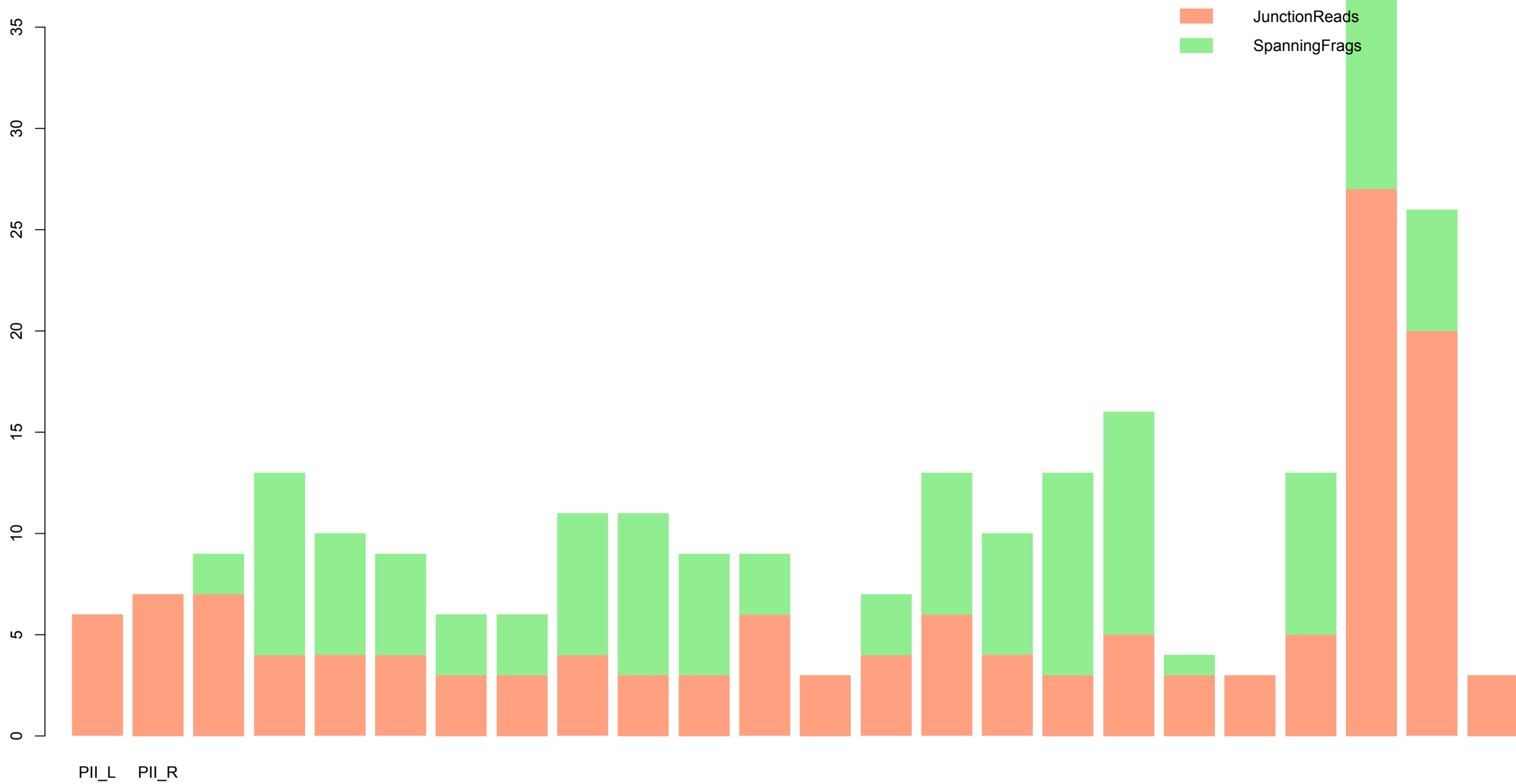

B

AP3D1--SLC6A8 junction read

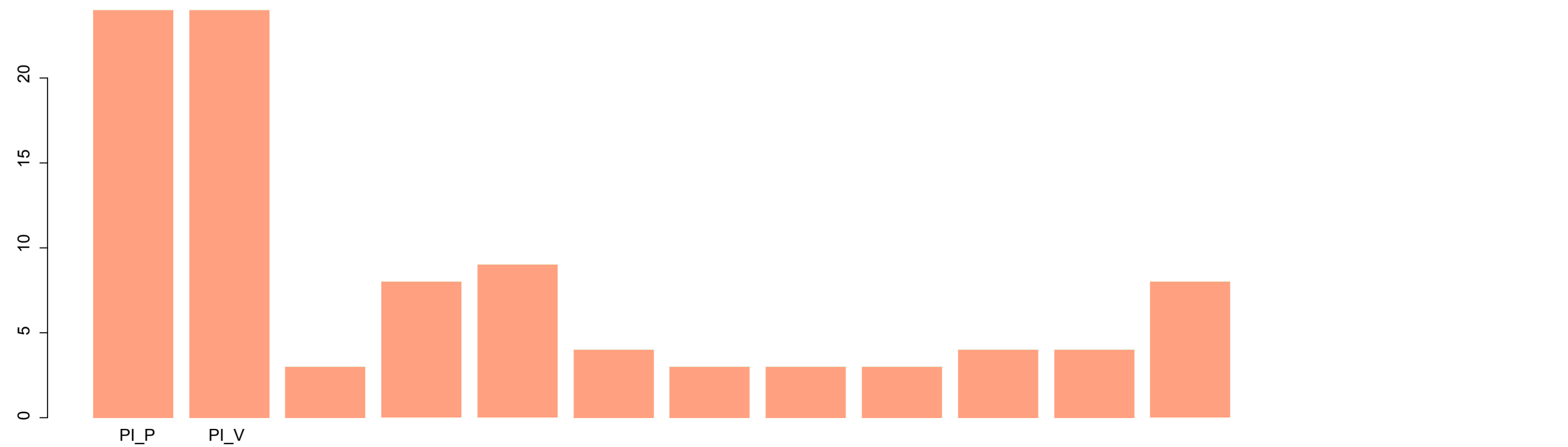

Supplement: Supplementary file 10 — Fig. S10. The junction reads of two known disease‐related fusion genes. (A) The junction reads of C15orf57‐CBX3. (B) The junction reads of AP3D1‐SLC6A8. [file MOL2-13-1214-s010.pdf]
