# Supplementary material for: The fusion landscape of hepatocellular carcinoma
Source: Mol Oncol. 2019 Apr 11;13(5):1214–25. doi: 10.1002/1878-0261.12479 (PMC6487730; doi:10.1002/1878-0261.12479)
Supplement: Supplementary file 2 — Fig. S2. The number of partner genes of each fusion genes in each samples. [file MOL2-13-1214-s002.pdf]

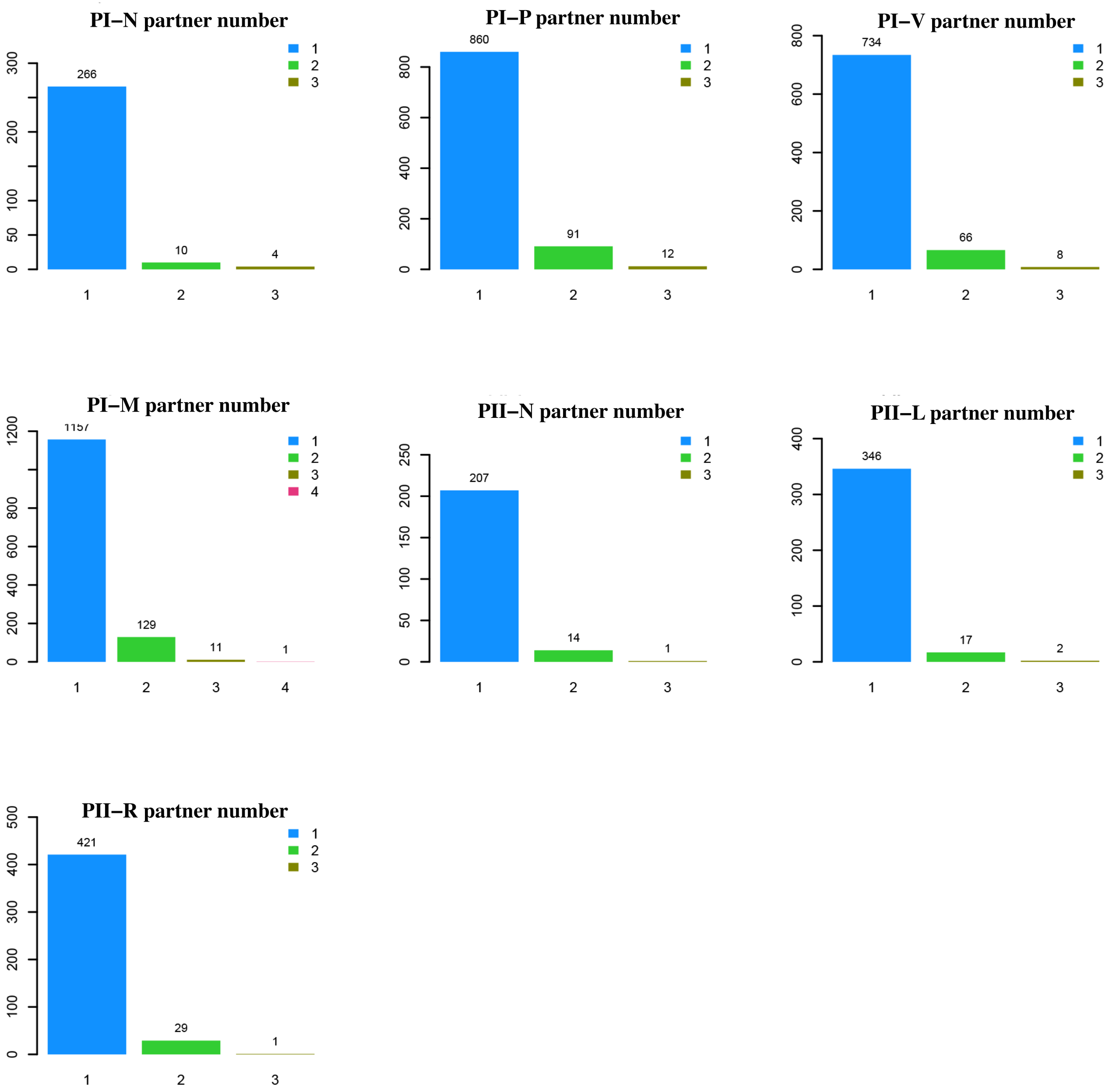
PI−N partner number
266
10
4
PI−P partner number
860
91
12
PI−V partner number
734
66
8
PI−M partner number
1157
129
11
1
PII−N partner number
207
14
1
PII−L partner number
346
17
2
PII−R partner number
421
29
1
