# Supplementary material for: The fusion landscape of hepatocellular carcinoma
Source: Mol Oncol. 2019 Apr 11;13(5):1214–25. doi: 10.1002/1878-0261.12479 (PMC6487730; doi:10.1002/1878-0261.12479)

A[#]

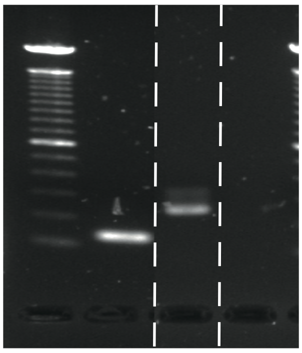

Marker GAPDH N57 C57

B

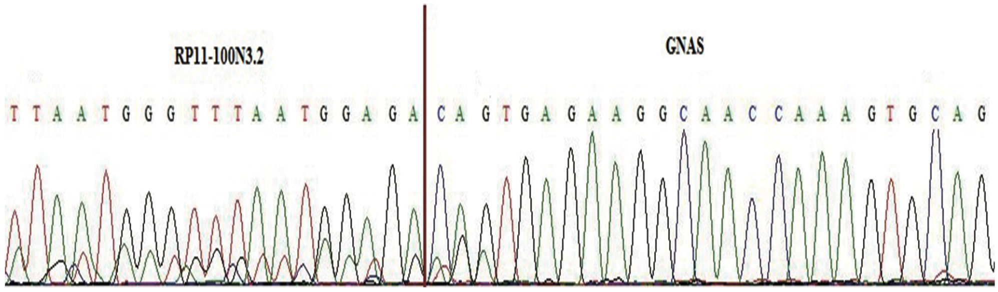

C

Patient	57	93	95	100	101	120	127	129	130	186	187
N	●	●	●	●	●		●	●		●	
C		●	●				●	●	●		●

Patient-P2			Patient-A			Patient-B		
P2N	P2L	P2R	AN	AC	AV	BN	BC1	BC2
●		●					●	

**recur-ratio**	Patient	Normal Sample	Tumor Sample
	0.86(12/14)	0.64(9/14)	0.47(8/17)

D

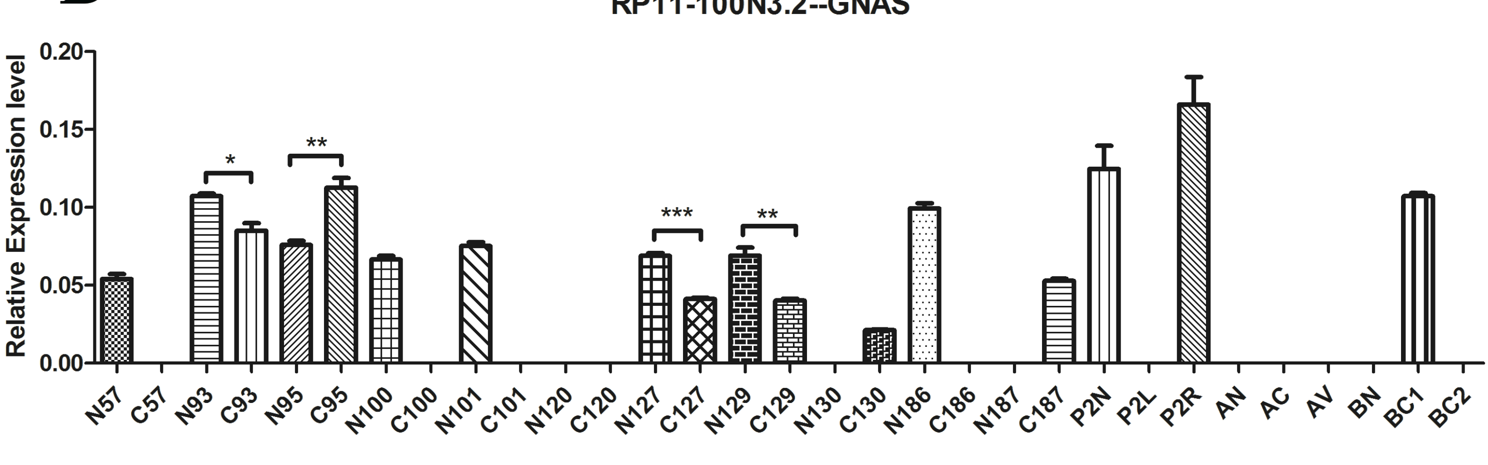

Supplement: Supplementary file 5 — Fig. S5. Details of RP11‐100N3.2–GNAS after experimental validation of the fusion transcripts by RT‐PCR, Sanger sequencing and qRT‐PCR. [file MOL2-13-1214-s005.pdf]
