# Supplementary material for: The fusion landscape of hepatocellular carcinoma
Source: Mol Oncol. 2019 Apr 11;13(5):1214–25. doi: 10.1002/1878-0261.12479 (PMC6487730; doi:10.1002/1878-0261.12479)

A#

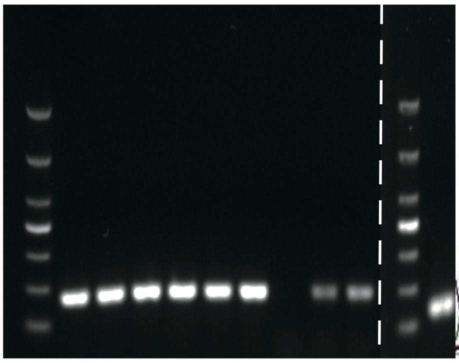

B

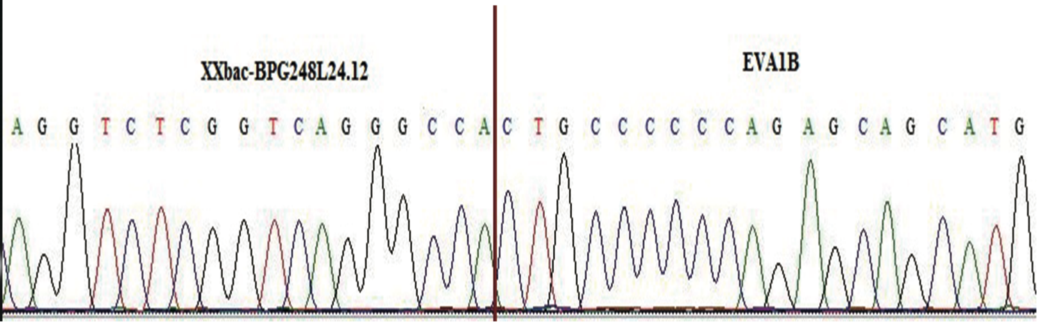

C

Patient	57	93	95	100	101	120	127	129	130	186	187
N	●	●	●	●	●	●	●	●	●	●	
C	●	●	●		●	●	●	●	●	●	●

Patient-P2			Patient-A			Patient-B		
P2N	P2L	P2R	AN	AC	AV	BN	BC1	BC2
●	●	●	●	●	●		●	●

recur-ratio	Patient	Normal Sample	Tumor Sample
	100%(14/14)	0.86(12/14)	0.94(16/17)

D

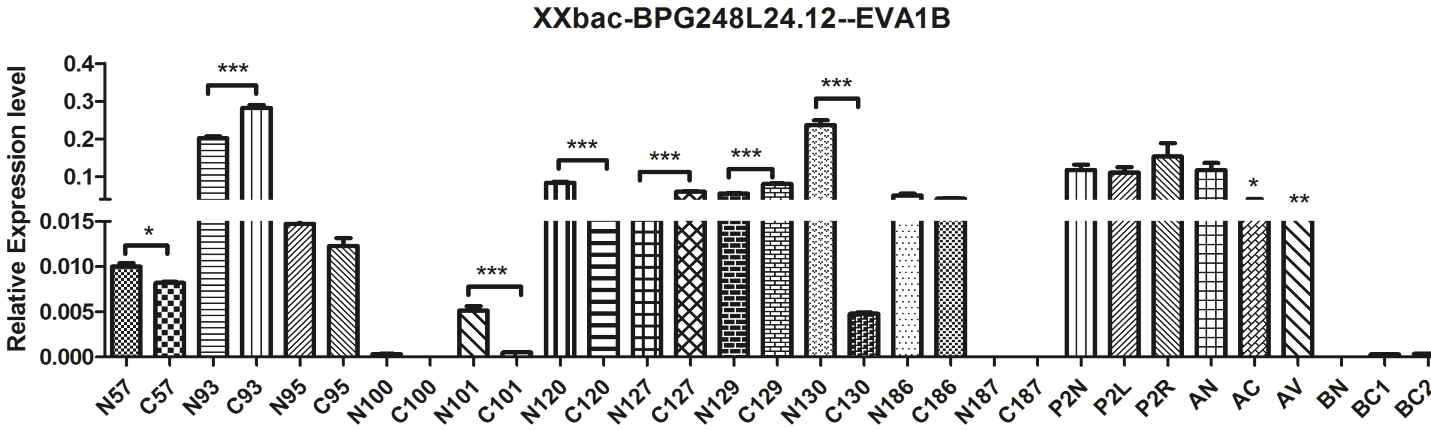

Supplement: Supplementary file 6 — Fig. S6. Details of XXbac‐BPG248L24.12–EVA1B after experimental validation of the fusion transcripts by RT‐PCR, Sanger sequencing and qRT‐PCR. [file MOL2-13-1214-s006.pdf]
