# Supplementary material for: The fusion landscape of hepatocellular carcinoma
Source: Mol Oncol. 2019 Apr 11;13(5):1214–25. doi: 10.1002/1878-0261.12479 (PMC6487730; doi:10.1002/1878-0261.12479)
Supplement: Supplementary file 7 — Fig. S7. Details of IGLV4‐69–IGLJ3 after experimental validation of the fusion transcripts by RT‐PCR and Sanger sequencing. [file MOL2-13-1214-s007.pdf]

A[#]

B

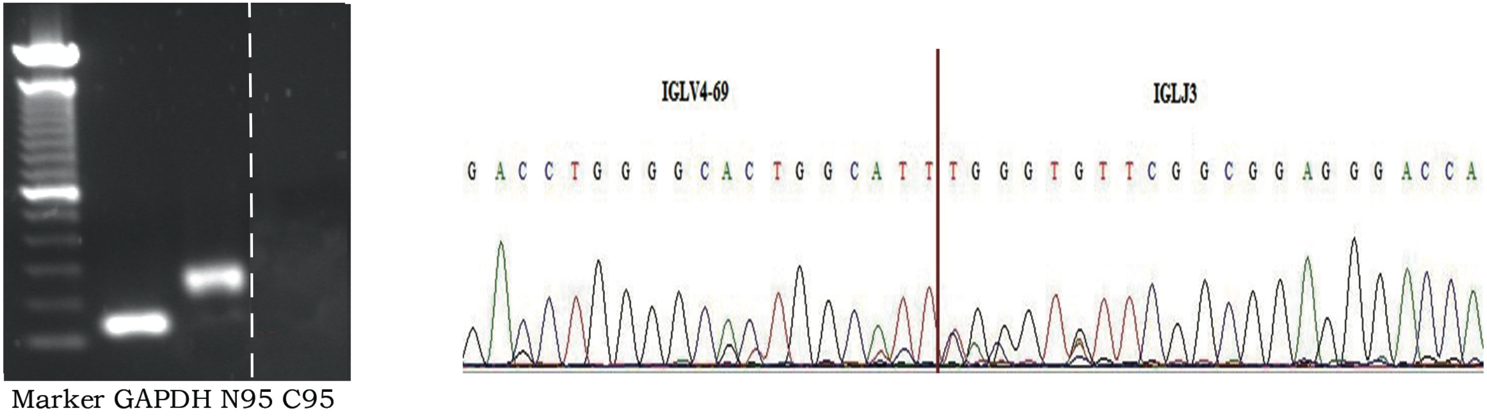

C

Patient	57	93	95	100	101	120	127	129	130	186	187
N			●	●							
C											

Patient-P2			Patient-A			Patient-B		
P2N	P2L	P2R	AN	AC	AV	BN	BC1	BC2
●				●	●			

**recur-ratio**	Patient	Normal Sample	Tumor Sample
	0.29(4/14)	0.21(3/14)	0.12(2/17)
