# Supplementary material for: The fusion landscape of hepatocellular carcinoma
Source: Mol Oncol. 2019 Apr 11;13(5):1214–25. doi: 10.1002/1878-0261.12479 (PMC6487730; doi:10.1002/1878-0261.12479)
Supplement: Supplementary file 8 — Fig. S8.The fusion events involved in known disease related fusion genes. (A) The breakpoint of known disease fusion C15orf57–CBX3. (B) The breakpoint of AP3D1—SLC6A8. [file MOL2-13-1214-s008.pdf]

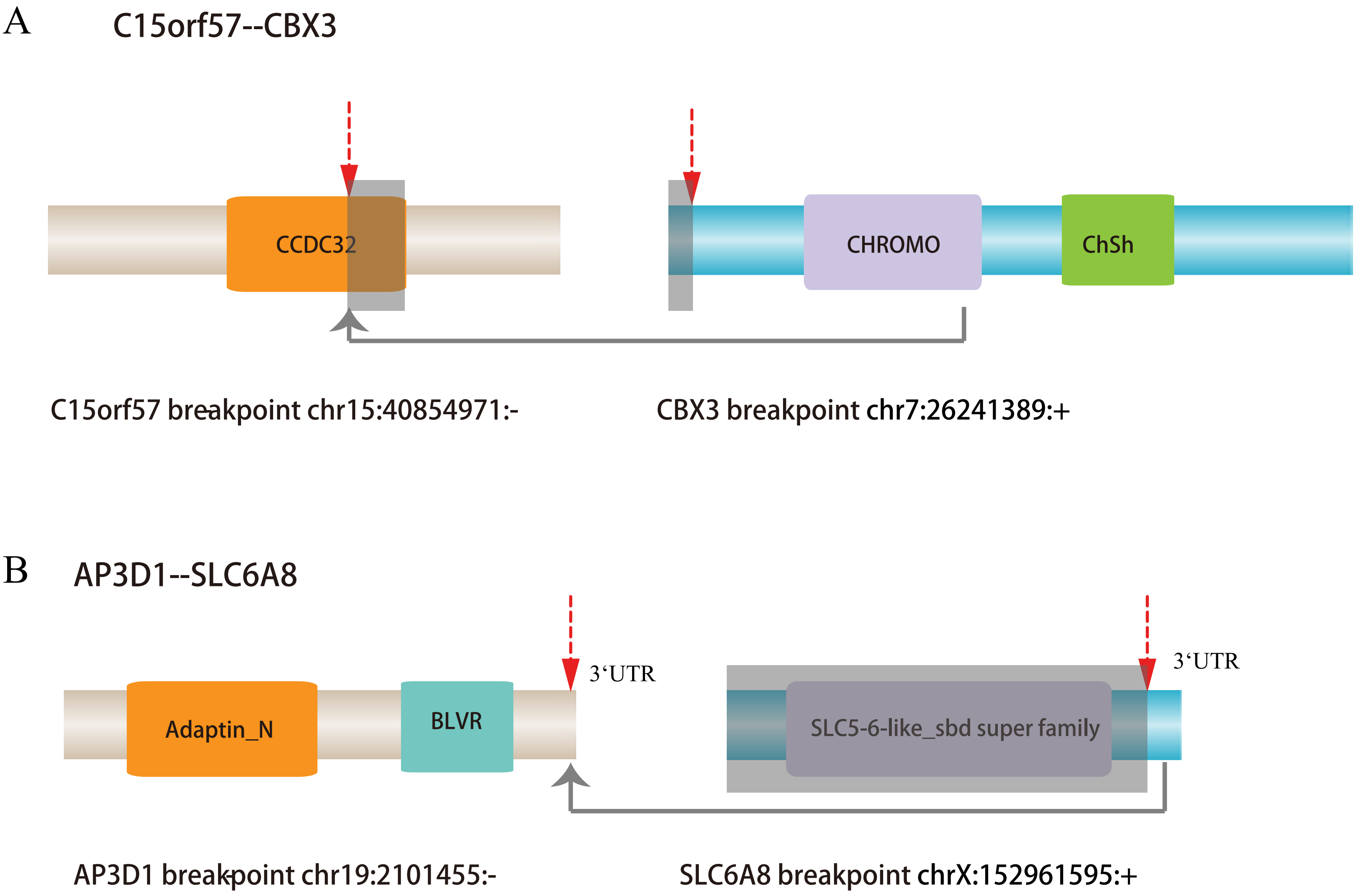

A
C15orf57--CBX3
CCDC32
CHROMO
ChSh
C15orf57 breakpoint chr15:40854971:-
CBX3 breakpoint chr7:26241389:+
B
AP3D1--SLC6A8
Adaptin_N
BLVR
3‘UTR
SLC5-6-like_sbd super family
3‘UTR
AP3D1 breakpoint chr19:2101455:-
SLC6A8 breakpoint chrX:152961595:+
