# Supplementary material for: The fusion landscape of hepatocellular carcinoma
Source: Mol Oncol. 2019 Apr 11;13(5):1214–25. doi: 10.1002/1878-0261.12479 (PMC6487730; doi:10.1002/1878-0261.12479)
Supplement: Supplementary file 9 — Fig. S9.The breakpoint of novel fusion events associated with HCC. (A) The breakpoint of DCUN1D3–GSG1L. (B) The breakpoint of SERPINA5–SERPINA9. [file MOL2-13-1214-s009.pdf]

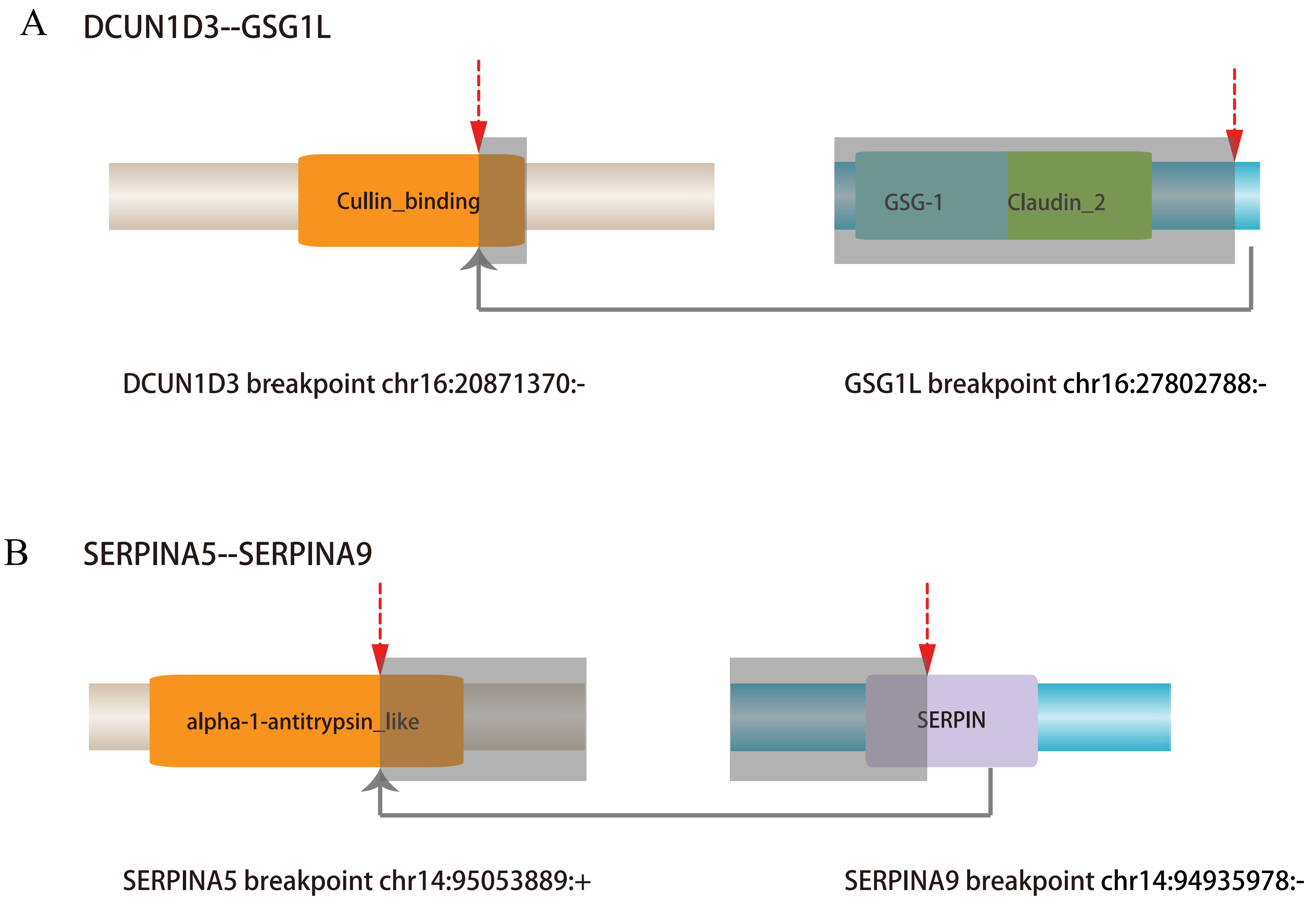
A
DCUN1D3--GSG1L
Cullin_binding
GSG-1
Claudin_2
DCUN1D3 breakpoint chr16:20871370:-
GSG1L breakpoint chr16:27802788:-
B
SERPINA5--SERPINA9
alpha-1-antitrypsin_like
SERPIN
SERPINA5 breakpoint chr14:95053889:+
SERPINA9 breakpoint chr14:94935978:-
