# Supplementary material for: The fusion landscape of hepatocellular carcinoma
Source: Mol Oncol. 2019 Apr 11;13(5):1214–25. doi: 10.1002/1878-0261.12479 (PMC6487730; doi:10.1002/1878-0261.12479)
Supplement: Supplementary file 11 — Table S1. The number of fusions with fusions occurring once and recurrent fusions supported by public HCC samples. [file MOL2-13-1214-s011.docx]

**Table S1.** The number of fusions with once fusions and recurrent fusions supported by public HCC samples.

|  | support | Non-support |
| --- | --- | --- |
| >=2 | 15 | 5 |
| ==1 | 23 | 2311 |
