# Supplementary material for: The fusion landscape of hepatocellular carcinoma
Source: Mol Oncol. 2019 Apr 11;13(5):1214–25. doi: 10.1002/1878-0261.12479 (PMC6487730; doi:10.1002/1878-0261.12479)
Supplement: Supplementary file 12 — Table S2. The breakpoint and junction reads of C15orf57–CBX3 across all samples where it occurred. [file MOL2-13-1214-s012.docx]

Table S2. The breakpoint and junction reads of C15orf57--CBX3 across all occurred samples.

|  | Fusion_name | JunctionReads | SpanningFrags | LeftBreakpoint | RightBreakpoint |
| --- | --- | --- | --- | --- | --- |
| PII_L | C15orf57--CBX3 | 6 | 0 | chr15:40854180:- | chr7:26241365:+ |
| PII_R | C15orf57--CBX3 | 7 | 0 | chr15:40854971:- | chr7:26241389:+ |
| SRR1946643_Tumor | C15orf57--CBX3 | 7 | 2 | chr15:40854180:- | chr7:26241365:+ |
| SRR1946646_Tumor | C15orf57--CBX3 | 3 | 9 | chr15:40854971:- | chr7:26241389:+ |
| SRR1946660_Tumor | C15orf57--CBX3 | 4 | 6 | chr15:40854971:- | chr7:26241389:+ |
| SRR1946661_Tumor | C15orf57--CBX3 | 4 | 5 | chr15:40854971:- | chr7:26241389:+ |
| SRR1946663_Tumor | C15orf57--CBX3 | 3 | 3 | chr15:40854971:- | chr7:26241389:+ |
| SRR1946670_Tumor | C15orf57--CBX3 | 3 | 3 | chr15:40854971:- | chr7:26241389:+ |
| SRR1946672_Tumor | C15orf57--CBX3 | 4 | 7 | chr15:40854180:- | chr7:26241365:+ |
| SRR1946680_Tumor | C15orf57--CBX3 | 3 | 8 | chr15:40854180:- | chr7:26241365:+ |
| SRR1946681_Tumor | C15orf57--CBX3 | 3 | 6 | chr15:40854971:- | chr7:26241389:+ |
| SRR1946682_Tumor | C15orf57--CBX3 | 6 | 3 | chr15:40854971:- | chr7:26241389:+ |
| SRR1946683_Tumor | C15orf57--CBX3 | 3 | 0 | chr15:40854180:- | chr7:26241365:+ |
| SRR1946685_Tumor | C15orf57--CBX3 | 4 | 3 | chr15:40854971:- | chr7:26241389:+ |
| SRR1946686_Tumor | C15orf57--CBX3 | 6 | 7 | chr15:40854971:- | chr7:26241389:+ |
| SRR1946687_Tumor | C15orf57--CBX3 | 4 | 6 | chr15:40854971:- | chr7:26241389:+ |
| SRR1946690_Tumor | C15orf57--CBX3 | 3 | 10 | chr15:40854180:- | chr7:26241365:+ |
| SRR1946691_Tumor | C15orf57--CBX3 | 4 | 11 | chr15:40854971:- | chr7:26241389:+ |
| SRR1186604_Tumor | C15orf57--CBX3 | 3 | 1 | chr15:40854971:- | chr7:26241389:+ |
| SRR1186610_Tumor | C15orf57--CBX3 | 3 | 0 | chr15:40854971:- | chr7:26241389:+ |
| SRR1946638_Normal | C15orf57--CBX3 | 5 | 8 | chr15:40854971:- | chr7:26241389:+ |
| SRR1946639_Normal | C15orf57--CBX3 | 27 | 10 | chr15:40854971:- | chr7:26241389:+ |
| SRR1946641_Normal | C15orf57--CBX3 | 8 | 6 | chr15:40854971:- | chr7:26241389:+ |
| SRR1186605_Normal | C15orf57--CBX3 | 3 | 0 | chr15:40854971:- | chr7:26241389:+ |
