# Supplementary material for: The fusion landscape of hepatocellular carcinoma
Source: Mol Oncol. 2019 Apr 11;13(5):1214–25. doi: 10.1002/1878-0261.12479 (PMC6487730; doi:10.1002/1878-0261.12479)
Supplement: Supplementary file 13 — Table S3. The breakpoint and junction reads of AP3D1–SLC6A8 across all samples where it occurred. [file MOL2-13-1214-s013.docx]

Table S3. The breakpoint and junction reads of AP3D1--SLC6A8 across all occurred samples.

|  | Fusion_name | JunctionReads | SpanningFrags | LeftBreakpoint | RightBreakpoint |
| --- | --- | --- | --- | --- | --- |
| PI_P | AP3D1--SLC6A8 | 24 | 0 | chr19:2101455:- | chrX:152961595:+ |
| PI_V | AP3D1--SLC6A8 | 24 | 0 | chr19:2101455:- | chrX:152961595:+ |
| SRR1946646_Tumor | AP3D1--SLC6A8 | 3 | 0 | chr19:2101455:- | chrX:152961595:+ |
| SRR1946650_Tumor | AP3D1--SLC6A8 | 8 | 0 | chr19:2101455:- | chrX:152961595:+ |
| SRR1946651_Tumor | AP3D1--SLC6A8 | 9 | 0 | chr19:2101455:- | chrX:152961595:+ |
| SRR1946658_Tumor | AP3D1--SLC6A8 | 4 | 0 | chr19:2101455:- | chrX:152961595:+ |
| SRR1946667_Tumor | AP3D1--SLC6A8 | 3 | 0 | chr19:2101455:- | chrX:152961595:+ |
| SRR1946672_Tumor | AP3D1--SLC6A8 | 3 | 0 | chr19:2101455:- | chrX:152961595:+ |
| SRR1946682_Tumor | AP3D1--SLC6A8 | 3 | 0 | chr19:2101455:- | chrX:152961595:+ |
| SRR1946684_Tumor | AP3D1--SLC6A8 | 4 | 0 | chr19:2101455:- | chrX:152961595:+ |
| SRR1186614_Tumor | AP3D1--SLC6A8 | 4 | 0 | chr19:2101455:- | chrX:152961595:+ |
| PI_N | AP3D1--SLC6A8 | 8 | 0 | chr19:2101455:- | chrX:152961595:+ |
