# Supplementary material for: The fusion landscape of hepatocellular carcinoma
Source: Mol Oncol. 2019 Apr 11;13(5):1214–25. doi: 10.1002/1878-0261.12479 (PMC6487730; doi:10.1002/1878-0261.12479)
Supplement: Supplementary file 14 — Table S4. The breakpoint and junction reads of DCUN1D3–GSG1L across all samples where it occurred. [file MOL2-13-1214-s014.pdf]

Table S4. The breakpoint and junction reads of DCUN1D3--GSG1L across all occurred samples.

| | New_fusion_name | JunctionReads | SpanningFrags | LeftBreakpoint | RightBreakpoint |
|---|---|---|---|---|---|
| PI_P | DCUN1D3--GSG1L | 25 | 12 | chr16:20871370:- | chr16:27802788:- |
| PI_V | DCUN1D3--GSG1L | 13 | 8 | chr16:20871370:- | chr16:27802788:- |
| PI_M | DCUN1D3--GSG1L | 26 | 15 | chr16:20871370:- | chr16:27802788:- |
