# Supplementary material for: The fusion landscape of hepatocellular carcinoma
Source: Mol Oncol. 2019 Apr 11;13(5):1214–25. doi: 10.1002/1878-0261.12479 (PMC6487730; doi:10.1002/1878-0261.12479)
Supplement: Supplementary file 15 — Table S5. The breakpoint and junction reads of SERPINA5–SERPINA9 across all samples where is occurred. [file MOL2-13-1214-s015.pdf]

Table S5. The breakpoint and junction reads of SERPINA5--SERPINA9 across all occurred samples.

	Fusion_name	JunctionReads	SpanningFrags	LeftBreakpoint	RightBreakpoint
PI_M	SERPINA5--SERPINA9	4	0	chr14:95053889:+	chr14:94935978:-
SRR1946652_Tumor	SERPINA5--SERPINA9	18	0	chr14:95053889:+	chr14:94935978:-
SRR1946656_Tumor	SERPINA5--SERPINA9	28	0	chr14:95053889:+	chr14:94935978:-
SRR1946659_Tumor	SERPINA5--SERPINA9	8	0	chr14:95053889:+	chr14:94935978:-
SRR1946671_Tumor	SERPINA5--SERPINA9	36	0	chr14:95053889:+	chr14:94935978:-
SRR1946675_Tumor	SERPINA5--SERPINA9	15	0	chr14:95053885:+	chr14:94935982:-
SRR1946677_Tumor	SERPINA5--SERPINA9	21	0	chr14:95053885:+	chr14:94935982:-
SRR1946681_Tumor	SERPINA5--SERPINA9	6	0	chr14:95053885:+	chr14:94935982:-
SRR1946688_Tumor	SERPINA5--SERPINA9	15	0	chr14:95053885:+	chr14:94935982:-
SRR1946691_Tumor	SERPINA5--SERPINA9	36	0	chr14:95053889:+	chr14:94935978:-
PI_N	SERPINA5--SERPINA9	5	0	chr14:95053889:+	chr14:94935978:-
