# Supplementary material for: The fusion landscape of hepatocellular carcinoma
Source: Mol Oncol. 2019 Apr 11;13(5):1214–25. doi: 10.1002/1878-0261.12479 (PMC6487730; doi:10.1002/1878-0261.12479)
Supplement: Supplementary file 16 — Table S6. The detail information of 43 candidate recurrent fusion genes. [file MOL2-13-1214-s016.pdf]

Table S6. The detail informations of 43 candidate recurrent fusion genes

Fusion_name	Novel fusion▲ (Y/N)	Interchrom/ Intrachrom	LeftBreakpoint	RightBreakpoint	FusionSample*	Tumor†	Normal‡	T/N&	T-N type#
IGLV1-51--IGLL5	N	intrachrom	chr22:22677323:+	chr22:23235962:+	PI_M	4	0	-	T >=1, N= 0
RABEP1--CLEC4D	N	interchrom	chr17:5288072:+	chr12:8674895:+	PII-L	4	0	-	T >=1, N= 0
DCUN1D3--GSG1L	Y	intrachrom	chr16:20871370:-	chr16:27802788:-	PI-P,PI-V,PI-M	3	0	-	T >=1, N= 0
CAPS--VMAC	Y	intrachrom	chr19:5911733:+	chr19:5910641:+	PI-P	3	0	-	T >=1, N= 0
RP11-476K15.1--CTD-2015H3.2	Y	intrachrom	chr18:1566525:+	chr18:1779860:+	PI-P,PII-R	3	0	-	T >=1, N= 0
FARS2--RP3-380B8.4	N	intrachrom	chr6:5613553:+	chr6:5870274:+	PII-R	3	0	-	T >=1, N= 0
BCL7C--LYNX1	N	interchrom	chr16:30903922:-	chr8:143845979:-	PI-V	2	0	-	T >=1, N= 0
PSPN--OPA3	N	intrachrom	chr19:6378873:-	chr19:46053264:-	PI-V,PII-L	2	0	-	T >=1, N= 0
MYL6--RAB3GAP1	N	interchrom	chr12:56553479:+	chr2:135872843:+	PII-R	2	0	-	T >=1, N= 0

AP3D1--SLC6A8	N	interchrom	chr19:2101455:-	chrX:152961595:+	PI-N,PI-P,PI-V	11	1	11	T >=1, N >=1
SERPINA5--SERPINA9	Y	intrachrom	chr14:95053889:+	chr14:94935978:-	PI-N,PI-M	10	1	10	T >=1, N >=1
TIMM23--BMS1P4/AGAP5	N	intrachrom	chr10:51606988:-	chr10:75473090:-	PII-N	26	4	6.5	T >=1, N >=1
C15orf57--CBX3	N	interchrom	chr15:40854180:-, chr15:40854971:-	chr7:26241365:+, chr7:26241389:+	PII-L,PII-R	20	4	5	T >=1, N >=1
RP11-100N3.2--GNAS	N	interchrom	chr11:56503838:-	chr20:57478583:+	PI-N,PI-V,PII-N	51	12	4.25	T >=1, N >=1
PPA2--AC005178.1	Y	interchrom	chr4:106290830:-	chr5:133056310:-	PII-L,PII-R	18	5	3.6	T >=1, N >=1
MT-TA--MT-TN	Y	intrachrom	chrM:5597:-, chrM:5607:-	chrM:5699:-, chrM:5706:-	PI-N,PII-R	30	9	3.3	T >=1, N >=1
XXbac-BPG248L24.12--EVA1B	Y	interchrom	chr6:31324886:+	chr1:36788655:-	PI-N,PI-P,PI-V,PI-M	9	3	3	T >=1, N >=1
IGLV4-69--IGLJ3	N	intrachrom	chr22:22385868:+	chr22:23247169:+	PI-M	3	1	3	T >=1, N >=1
IGKV1-39--AC096579.7	Y	intrachrom	chr2:89619384:-	chr2:89161435:-	PII-N	3	1	3	T >=1, N >=1

HLA-DPA1--FBXO31	N	interchrom	chr6:33037420:-	chr16:87425701:-	PI-N,PI-P,PI-M	17	6	2.83	T >=1, N >=1
RP11-672L10.2--ALDH4A1	Y	interchrom	chr18:906381:-	chr1:19202915:-	PI-N,PI-P,PI-V,PI-M, PII-L,PII-R	8	3	2.67	T >=1, N >=1
CDH23--HLA-DPB1	N	interchrom	chr10:73491961:-	chr6:33048697:+	PI-P,PI-M,PII-N	23	9	2.56	T >=1, N >=1
IGKV4-1--IGKJ1	Y	intrachrom	chr2:89185668:+	chr2:89161435:-	PI-N	2	1	2	T >=1, N >=1
IGLV1-44--BMS1P20	N	intrachrom	chr22:22735523:+	chr22:2267713 3:+	PI-N	2	1	2	T >=1, N >=1
HLA-DPB2--HLA-DRB1	N	intrachrom	chr6:33095850:+	chr6:32549358:-	PI-N,PI-P,PI-M	2	1	2	T >=1, N >=1
TIMP1--ZNF469	N	interchrom	chrX:47444365:+	chr16:88494281:+	PI-M,PII-N,PII-R	2	1	2	T >=1, N >=1
IGKV4-1--IGKJ2	Y	intrachrom	chr2:89185668:+	chr2:89161074:-	PII-R	5	3	1.67	T >=1, N >=1
TTC39B--OPN3	Y	interchrom	chr9:15170192:-	chr1:241756815:-	PI-M	12	8	1.5	T >=1, N >=1
HRG--RPUSD1	Y	interchrom	chr3:186395245:+, chr3:186395215:+	chr16:836248:-, chr16:836248:-	PI-P,PI-M	6	5	1.2	T >=1, N >=1

MCC--RPL23AP79	N	interchrom	chr5:112360726:-	chr19:59094032:+	PI-M,PII-L	6	6	1	T >=1, N >=1
IGKV3-20--IGKJ2	N	intrachrom	chr2:89442058:-	chr2:89161074:-	PII-N	2	2	1	T >=1, N >=1
IGKV3-15--IGKJ1	Y	intrachrom	chr2:89384674:-	chr2:89161432:-	PI-N	1	1	1	T >=1, N >=1
CTSS--TAPBP	N	interchrom	chr1:150705079:-	chr6:33268494:-	PI-N	1	1	1	T >=1, N >=1
TTYH2--ADIRF	Y	interchrom	chr17:72227130:+	chr10:88730313:+	PI-P,PII-N	1	1	1	T >=1, N >=1
ARHGDIA--LINC00511	N	intrachrom	chr17:79826258:-	chr17:70379417:-	PI-M	1	1	1	T >=1, N >=1
RP5-940J5.9--GAPDHP40	Y	interchrom	chr12:6647307:-	chr5:159377586:-	PII-N	1	1	1	T >=1, N >=1
NIPA2--POLA1	N	interchrom	chr15:23005214:-	chrX:24721385:+	PII-L	1	1	1	T >=1, N >=1
MED25--MAT1A	N	interchrom	chr19:50333190:+	chr10:82031941:-	PII-N,PII-L	4	5	0.8	T >=1, N >=1
RP11-96H19.1--RP11-446N19.1	Y	intrachrom	chr12:46781755:+	chr12:47046173:+	PI-N,PI-P,PII-N	4	8	0.5	T >=1, N >=1

IGKV2-30--AC096579.7	**Y**	intrachrom	chr2:89544265:-	chr2:89161074:-	PII-N	1	2	0.5	T >=1, N >=1
OTUD6A--CTSB	**N**	interchrom	chrX:69283986:+	chr8:11700207:-	PI-N	1	3	0.33	T >=1, N >=1
IGKV2-30--IGKJ1	**Y**	intrachrom	chr2:89544265:-	chr2:89161435:-	PI-N	1	5	0.2	T >=1, N >=1
C3orf27--SLC22A1	**N**	interchrom	chr3:128294109:-	chr6:160579548:+	PI-N	1	9	0.11	T >=1, N >=1
