# Supplementary material for: The fusion landscape of hepatocellular carcinoma
Source: Mol Oncol. 2019 Apr 11;13(5):1214–25. doi: 10.1002/1878-0261.12479 (PMC6487730; doi:10.1002/1878-0261.12479)
Supplement: Supplementary file 17 — Table S7. The primer sequences for candidate recurrent fusion genes and internal control gene(GAPDH). [file MOL2-13-1214-s017.docx]

Table S7. The primer sequences for candidate recurrent fusion genes and internal control gene（GAPDH）.

| **Item** | **Gene** | **Forward** | **Reverse** |
| --- | --- | --- | --- |
| **fusion gene** |  |  |  |
|  | *IGLV1-51--IGLL5* | ATTACTGCGGAACATGGGATA | GTTTGGAGGGTTTGGTGGT |
|  | *RABEP1--CLEC4D* | GCATCAGCAGCAGCCAGAG | ACGGTCAGCTTGGTCCCTC |
|  | *DCUN1D3--GSG1L* | CTGCATCGGGAAATAGCCA | ACACCCAGTGCCCCAAGAC |
|  | *CAPS--VMAC* | CACCCTGCCACCACTGCAC | CTCTGGGCCTCTGTTTGTT |
|  | *RP11-476K15.1--CTD-2015H3.2* | GTGCTTGCTTGGACCTTTC | TTTTCACATGCTGGATGCC |
|  | *FARS2--RP3-380B8.4* | TGGTGCTCAAGACCGAATC | AGAGGGCAACCAGAATGAA |
|  | *BCL7C--LYNX1* | CAGCAACCAGAGTTTCCATTCG | ACAAGCGGTGCTGGACGGT |
|  | *PSPN--OPA3* | GTGATCTGCCTGCCTCGTC | CCCTCTGCTCCAGCTTCTC |
|  | *MYL6--RAB3GAP1* | AAGACCAGACCGCAGAGTT | ATCCAGCAGACCTGATAAGTGAG |
|  | *AP3D1--SLC6A8* | TGGTGACTGGGTCTTGGGATC | TGGCAAAGGGACAGCGTGGG |
|  | *SERPINA5--SERPINA9* | CTCTTCCTCCTCTTGTGCC | GAGAATCTGGGTCTTGGTG |
|  | *TIMM23--BMS1P4/AGAP5* | GGGGAAGCGGCAACAAA | GGAGCCAGACAGCAGCACA |
|  | *C15orf57--CBX3* | TGTTCCTGTCTGCCAAATC | GCTATTATTCACCGCCTCC |
|  | *RP11-100N3.2--GNAS* | TGCTGGAGAATCTGGTAAAG | GGGAGGGAAGTCAAAGTCA |
|  | *PPA2--AC005178.1* | TAGCCCTTTCCGTTGCACTC | TGCTTCATCCTTGGAAACACTG |
|  | *MT-TA--MT-TN* | GATTTGCGTTCAGTTGATG | GACCAATGGGACTTAAACC |
|  | *XXbac-BPG248L24.12--EVA1B* | CGTCGCTGTCGAACCTCACG | GGGCATCCATGCTGCTCTGG |
|  | *IGLV4-69--IGLJ3* | GCATCAGCAGCAGCCAGAG | ACGGTCAGCTTGGTCCCTC |
|  | *IGKV1-39--AC096579.7* | TCAATCGCCCTCTGCCTCT | CTCCGCCGAACACCCAAAT |
|  | *HLA-DPA1--FBXO31* | GGCATCTGGAGGAGTTTGG | AAGGCACAGCTTCAGAGGTTA |
|  | *RP11-672L10.2--ALDH4A1* | TGGGGTCTTTCTGGATATTGC | CACAAAGTAGCCCACGGAGTC |
|  | *CDH23--HLA-DPB1* | GGGAAGTTTGAGATTGACGA | ATGCTGCCTGGGTAGAAAT |
|  | *IGKV4-1--IGKJ1* | CAAGTCCAGCCAGAGTGTT | GATTTCCACCTTGGTCCCT |
|  | *IGLV1-44--BMS1P20* | CTCACCCTCCTCACTCACT | GGGTCGCTTATTATTGTCA |
|  | *HLA-DPB2--HLA-DRB1* | GGGAGGAATACGTGCATTTTG | GGCTCGCTTCCGTTCCATA |
|  | *TIMP1--ZNF469* | GCTTCTGGCATCCTGTTGTTG | GGGTTGGGCCTCAGTCCTT |
| **internal control gene** |  |  |  |
|  | *GAPDH* | TCAAGGCTGAGAACGGGAAG | GTGAAGACGCCAGTGGACT |
