# Supplementary material for: The fusion landscape of hepatocellular carcinoma
Source: Mol Oncol. 2019 Apr 11;13(5):1214–25. doi: 10.1002/1878-0261.12479 (PMC6487730; doi:10.1002/1878-0261.12479)
Supplement: Supplementary file 18 — Data S1. The sequences for validation recurrent fusion genes. [file MOL2-13-1214-s018.docx]

**Supplementary data A. The sequences for validation recurrent fusion genes**

**C****1****5orf57--****C****BX3**

>C15orf57__chr15:40854971:-__CBX3__chr7:26241389:+__ENST00000560305.1__ENST00000396386.2__292

AGAGCTGAGCCAAGCGTTTACTGGGCAGCTGTTACGCTCAGATTCCAAATGAAAATGTTTGAGAGCGCTGACTCTACAGCCACAAGATCTGGCCAGGATCTCTGGGCTGAAATTTGTTCCTGTCTGCCAAATCCTGAACAAGAAGATGGTGCCAACAATGCATTCTCAGACTCCTTTGTGGATTCTTGCCCTGAAGGTGAAGGCCAGAGGGAGGTGGCTGACTTTGCTGTCCAGCCAGCTGTAAAGCCTTGGGCTCCCTTGCAGGATTCAGAAGTGTATTTAGCATCTCTA**GG**CGCGGAGGGCCGGAGACGCTGCAGACCCGCGACCCGGAGCAGCTCGGAGGCGGTGAATAATAGCTCTTCAAGTCTGCAATAAAAAATGGCCTCCAACAAAACTACATTGCAAAAAATGGGAAAAAAACAGAATGGAAAGAGTAAAAAAGTTGAAGAGGCAGAGCCTGAAGAATTTGTCGTGGAAAAAGTACTAGATCGACGTGTAGTGAATGGGAAAGTGGAATATTTCCTGAAGTGGAAGGGATTTACAGATGCTGACAATACTTGGGAACCTGAAGAAAATTTAGATTGTCCAGAATTGATTGAAGCGTTTCTTAACTCTCAGAAAGCTGGCAAAGAAAAAGATGGTACAAAAAGAAAATCTTTATCTGACAGTGAATCTGATGACAGCAAATCAAAGAAGAAAAGAGATGCTGCTGACAAACCAAGAGGATTTGCCAGAGGTCTTGATCCTGAAAGAATAATTGGTGCCACAGACAGCAGTGGAGAATTGATGTTTCTCATGAAATGGAAAGATTCAGATGAGGCAGACTTGGTGCTGGCGAAAGAGGCAAATATGAAGTGTCCTCAAATTGTAATTGCTTTTTATGAAGAGAGACTAACTTGGCATTCTTGTCCAGAAGATGAAGCTCAATAATTGTTCACATTGTTCTTTTATATATATTTATATATATATATAAAAATTGGGTCTTAGATTTTGATTTACTAGTGTGACAAAATAACTACATCCTAATGAAAATCAAGTTTGATATGTTTGTTTTGAAAGTAGCGTTGGAAGAGTTGTTGGGGGTTTTTTGCATCCATAGCACTGGTTACTTTGAACAAATAAATAAAAGCTTTCTGTAGTTGCTTCCTTTATCAGAAAAGAACATTTGATACCATGGTATATCATTTCCTCTTCATTAAAGAACAGCTTTTCTAAATGTTGGGGGAAATGTCCATAGTCATTACTCAGTCAAAACTTGTGTTCTCATGAGCCTAAGGACCATTCTAGATTTATTACGTGTTTTTTGTGTGTGTGTGTGTGTGTGTGTGTGTGTGTATCCATAAAATGCATATGTAAATTTTTTTTTGTTTTTAAGCATTCACCCAAACAAAAAAATCACAGGTAAACCCATGTTTCTGAGATGCCATTATTCCAAGCAAAATAAGAGATAATCCCTTCAAGTTAAATTGAAAATTTTCCTGAAACCATACATTTCAAGTGAAATAAGTAATTCTAGATAGGACAATTTAAATTGGATAATTTTAAAGTGTCTATAATTGCAGTGGTTTATTTGCAAAATTCCTAAAAGGAAAAATTTTATCACTGCCATCACAGCAGGTTTCCTCATCCAGATGAGGAAACTAGACAAATGCTAGTGTGTTTTAACTAGCTAAACAAAACTAAGTTAAATGAACATTTAAAAGTTTCCCTAGCGGGCCATTCCTTAGCAAAATGTTGGAATCCCTGTTGCTACATTGACTAAAAGGTCATGATGAATGGAATATGTAAGACTTGGCTCATAGAAACCTAATCAGATGGTTAGAGGTGTTGGCAGTTTAGGACCTGCTGTCATAAATGTGTGAACAACCTTTTGTAACCTAACCTATTGACCTGCATGTTTTTTCTTTACCCCAATTCATTACATGGAGGCTCAATCTTGAGTTTGCTTTACTGGTTCAGCAAAAGCCAGGAAGAACAACTTTGTAGTAATCAAAATGTTATCCAACTGTATATTGTTTACTTTATTGTAAATACTGGTGAACAGTGGTTAATAAATAGTTTTATATTCCTTTA

**IGLV1-51--IGLL5**

>IGLV1-51__chr22:22677323:+__IGLL5__chr22:23235962:+__ENST00000390290.2__ENST00000526893.1__387

TGAGCGCAGAAGGCAGGACTCGGGACAATCTTCATCATGACCTGCTCCCCTCTCCTCCTCACCCTTCTCATTCACTGCACAGGGTCCTGGGCCCAGTCTGTGTTGACGCAGCCGCCCTCAGTGTCTGCGGCCCCAGGACAGAAGGTCACCATCTCCTGCTCTGGAAGCAGCTCCAACATTGGGAATAATTATGTATCCTGGTACCAGCAGCTCCCAGGAACAGCCCCCAAACTCCTCATTTATGACAATAATAAGCGACCCTCAGGGATTCCTGACCGATTCTCTGGCTCCAAGTCTGGCACGTCAGCCACCCTGGGCATCACCGGACTCCAGACTGGGGACGAGGCCGATTATTACTGCGGAACATGGGATAGCAGCCTGAGTGC**TT**ATGTCTTCGGAACTGGGACCAAGGTCACCGTCCTAGGTCAGCCCAAGGCCAACCCCACTGTCACTCTGTTCCCGCCCTCCTCTGAGGAGCTCCAAGCCAACAAGGCCACACTAGTGTGTCTGATCAGTGACTTCTACCCGGGAGCTGTGACAGTGGCCTGGAAGGCAGATGGCAGCCCCGTCAAGGCGGGAGTGGAGACCACCAAACCCTCCAAACAGAGCAACAACAAGTACGCGGCCAGCAGCTACCTGAGCCTGACGCCCGAGCAGTGGAAGTCCCACAGAAGCTACAGCTGCCAGGTCACGCATGAAGGGAGCACCGTGGAGAAGACAGTGGCCCCTACAGAATGTTCATAGGTTCCCAACTCTAACCCCACCCACGGGAGCCTGGAGCTGCAGGATCCCAGGGGAGGGGTCTCTCTCCCCATCCCAAGTCATCCAGCCCTTCTCCCTGCACTCATGAAACCCCAATAAATATCCTCATTGAC

**IGLV4-69--IGLJ3**

>IGLV4-69__chr22:22385868:+__IGLJ3__chr22:23247169:+__ENST00000390282.2__ENST00000390324.2__417

AGGGTGGGTAAGAAATACCTGCAACTGTCAGCCTCAGCAGAGCTCTGGGGAGTCTGCACCATGGCTTGGACCCCACTCCTCTTCCTCACCCTCCTCCTCCACTGCACAGGGTCTCTCTCCCAGCTTGTGCTGACTCAATCGCCCTCTGCCTCTGCCTCCCTGGGAGCCTCGGTCAAGCTCACCTGCACTCTGAGCAGTGGGCACAGCAGCTACGCCATCGCATGGCATCAGCAGCAGCCAGAGAAGGGCCCTCGGTACTTGATGAAGCTTAACAGTGATGGCAGCCACAGCAAGGGGGACGGGATCCCTGATCGCTTCTCAGGCTCCAGCTCTGGGGCTGAGCGCTACCTCACCATCTCCAGCCTCCAGTCTGAGGATGAGGCTGACTATTACTGTCAGACCTGGGGCACTGGCAT**TT**GGGTGTTCGGCGGAGGGACCAAGCTGACCGTCCTAG

**RP11-100N3.2--GNAS**

>RP11-100N3.2__chr11:56503838:-__GNAS__chr20:57478583:+__ENST00000525668.1__ENST00000371075.3__101

CGTGCCATGGACCGCCTGCTACTCCTGGGTGCTGGAGAATCTGGTAAAGGCACCAATGTGAAGCAGATGAGGATCCTACATGTTAATGGGTTTAATGGAG**AC**AGTGAGAAGGCAACCAAAGTGCAGGACATCAAAAACAACCTGAAAGAGGCGATTGAAACCATTGTGGCCGCCATGAGCAACCTGGTGCCCCCCGTGGAGCTGGCCAACCCCGAGAACCAGTTCAGAGTGGACTACATCCTGAGTGTGATGAACGTGCCTGACTTTGACTTCCCTCCCGAATTCTATGAGCATGCCAAGGCTCTGTGGGAGGATGAAGGAGTGCGTGCCTGCTACGAACGCTCCAACGAGTACCAGCTGATTGACTGTGCCCAGTACTTCCTGGACAAGATCGACGTGATCAAGCAGGCTGACTATGTGCCGAGCGATCAGGACCTGCTTCGCTGCCGTGTCCTGACTTCTGGAATCTTTGAGACCAAGTTCCAGGTGGACAAAGTCAACTTCCACATGTTTGACGTGGGTGGCCAGCGCGATGAACGCCGCAAGTGGATCCAGTGCTTCAACGATGTGACTGCCATCATCTTCGTGGTGGCCAGCAGCAGCTACAACATGGTCATCCGGGAGGACAACCAGACCAACCGCCTGCAGGAGGCTCTGAACCTCTTCAAGAGCATCTGGAACAACAGATGGCTGCGCACCATCTCTGTGATCCTGTTCCTCAACAAGCAAGATCTGCTCGCTGAGAAAGTCCTTGCTGGGAAATCGAAGATTGAGGACTACTTTCCAGAATTTGCTCGCTACACTACTCCTGAGGATGCTACTCCCGAGCCCGGAGAGGACCCACGCGTGACCCGGGCCAAGTACTTCATTCGAGATGAGTTTCTGAGGATCAGCACTGCCAGTGGAGATGGGCGTCACTACTGCTACCCTCATTTCACCTGCGCTGTGGACACTGAGAACATCCGCCGTGTGTTCAACGACTGCCGTGACATCATTCAGCGCATGCACCTTCGTCAGTACGAGCTGCTCTAAGAAGGGAACCCCCAAATTTAATTAAAGCCTTAAGCACAATTAATTAAAAGTGAAACGTAATTGTACAAGCAGTTAATCACCCACCATAGGGCATGATTAACAAAGCAACCTTTCCCTTCCCCCGAGTGATTTTGCGAAACCCCCTTTTCCCTTCAGCTTGCTTAGATGTTCCAAATTTAGAAAGCTTAAGGCGGCCTACAGAAAAAGGAAAAAAGGCCACAAAAGTTCCCTCTCACTTTCAGTAAAAATAAATAAAACAGCAGCAGCAAACAAATAAAATGAAATAAAAGAAACAAATGAAATAAATATTGTGTTGTGCAGCATTAAAAAAAATCAAAATAAAAATTAAATGTGAGCAAAGAA

**RP11-476K15.1--CTD-2015H3.2**

>RP11-476K15.1__chr18:1566525:+__CTD-2015H3.2__chr18:1779860:+__ENST00000583163.1__ENST00000580524.1__114

GTGCCGGGATTACAAGCGTGAGCCACCATTCCCGGCCCAAAGAGGAGAAGAAGTATGAAGTGCTTGCTTGGACCTTTCTACTGTGCTTATTATTCTGTCTTCTCCTCATGGCA**AC**ATGTGCTGTTCTCTCTGTGGACATGTCTCTGGCTACCTGAAGGCATCCAGCATGTGAAAAAACAACTCAGACCACACATTTTCCACCTATGTTCTT

**XXbac-BPG248L24.12--EVA1B**

>XXbac-BPG248L24.12__chr6:31324886:+__EVA1B__chr1:36788655:-__ENST00000603274.1__ENST00000270824.1__463

ATGGGGAGTCGTGACCTGCGCCCCGGGCCGGGGTCACTCACCGGCCTCGCTCTGGTTGTAGTAGCCGCGCAGGTTCCGCAGGCTCTCTCGGTCAGTCTGTGCCTGGGCCTTGTAGATCTGTGTGTTCCGGTCCCAATACTCCGGCCCCTCCTGCTCTATCCACGGCGCCCGCGGCTCCTCTCTCGGACTCGCGGCGTCGCTGTCGAACCTCACGAACTGGGTGTCGTCCACGTAGCCCACTGAGATGAAGCGGGGCTCCCCGCGGCCGGGCCGGGACACGGAGGTGTAGAAATACCTCATGGAGTGGGAGCCTGGGGGTGAGGAGGGGCTGAGACCCGCCCGACCCTCCTCCCGGCGCGGCTCCTCAGGTCCTGCGCCCCCGCCTGCGGTCCCCTCGCTCCTCCCGGCAGAGGCCATTTCCCTCCCGACCCGCACTCACCGGCCCAGGTCTCGGTCAGGGCC**AC**TGCCCCCCAGAGCAGCATGGATGCCCCGCGAAGGGACATGGAGTTGCTCAGCAACAGCCTGGCTGCCTACGCGCACATCCGCGCCAACCCCGAGAGCTTCGGCCTCTACTTCGTGCTGGGCGTCTGCTTCGGCCTGCTGCTCACCCTCTGCCTGCTCGTCATCAGCATCTCGTGGGCGCCCCGCCCGCGGCCCCGGGGCCCGGCTCAGCGCCGGGACCCCCGCAGCAGCACCCTGGAGCCCGAGGACGACGACGAGGACGAGGAGGACACGGTGACTCGGCTGGGCCCCGACGACACGCTGCCGGGCCCCGAGCTGTCCGCAGAGCCGGACGGGCCCCTCAACGTCAACGTCTTCACGTCGGCGGAGGAGCTGGAGCGGGCGCAGCGGCTGGAGGAGCGCGAACGGATCCTGCGGGAGATCTGGCGCACCGGGCAGCCGGACCTGCTGGGCACAGGCACGCTGGGGCCCAGCCCCACGGCCACGGGCACCCTGGGCCGCATGCACTATTACTGATGGGCCCCGGCTCCCGCTGCAAGGCGCTCGGGGTACCGGACCTGCACATGAGCTCAGAGCTACCCCACACCTTCGGACTGCCTCGGCCCCCACAGCTCCCAGGTGCTACTGGGCGTGGACCGCCACCCCCTGAGAGGCTCCCTTCCCCAGTCCTGCCAGAAGACCCCGGGGGCGGGGAGGGGGCAGCATGCAGGGTCCCCACTCCCTCTCTGGGGTCGATGAAGAGGTGAAGTGACCAAATGAAAGAAAGCTGCATTCTCAGTG
