# Supplementary material for: The fusion landscape of hepatocellular carcinoma
Source: Mol Oncol. 2019 Apr 11;13(5):1214–25. doi: 10.1002/1878-0261.12479 (PMC6487730; doi:10.1002/1878-0261.12479)
Supplement: Supplementary file 19 [file MOL2-13-1214-s019.docx]

**Supporting information**

**Table S1.** The number of fusions with once fusions and recurrent fusions supported by public HCC samples.

**Table S2.** The breakpoint and junction reads of C15orf57--CBX3 across all occurred samples.

**Table S3.** The breakpoint and junction reads of AP3D1--SLC6A8 across all occurred samples.

**Table S4.** The breakpoint and junction reads of DCUN1D3--GSG1L across all occurred samples.

**Table S5.** The breakpoint and junction reads of SERPINA5--SERPINA9 across all occurred samples.

**Table S6.** The detail informations of 43 candidate recurrent fusion genes.

**^▲^**Y means the fusion gene was a novel and recurrent fusion that has not been previously annotated to diseases, N means the fusion gene has been annotated to diseases.

**^*^**The fusion samples which occurred in our HCC samples

**^†^**The number of tumor samples in our HCC samples and public 79 samples

**^‡^**The number of normal/adjacent noncancerous liver samples in our HCC samples and public 79 samples

**^&^** The ratio of Number-Tumor and Number-Normal

**^#^** The candidate recurrent fusion genes are divided into two types. T >=1,N= 0 : The gene fusion which occurred only in tumor samples(including our HCC samples and public 79 samples)；T >=1,N >=1：The gene fusion which occurred in both tumor and normal samples(including our HCC samples and public 79 samples).

**Table S7.** The primer sequences for candidate recurrent fusion genes and internal control gene（GAPDH）.

**Figure S1.** (A) The transcriptome component of liver tissue the classification of lncRNAs. (B-C) The patient-specific transcriptome.

**Figure S2.** The number of partner genes of each fusion genes in each samples.

**Figure S3.** Kinase gene fusions in HCC.


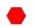
represents kinase gene;
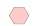
represents ordinary protein-coding gene;
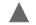
represents pseudogenes;
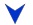
represents lncRNA genes;
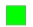
represents other genes;
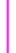
represents fusion event occurred in tumor tissue;
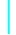
represents fusion event occurred in adjacent non-tumor tissue.

**F****igure S4.** Details of IGLV1-51--IGLL5 after experimental validation of the fusion transcripts by RT-PCR, Sanger sequencing and Quantitative Real-time PCR.

(A-B) The electrophoretic result and sequencing data for RT-PCR product with fusion gene IGLV1-51--IGLL5. (C) Verified samples for the existence fusion IGLV1-51—IGLL5. Recur-ratio shows the ratio of the verified sample comparing with the total number in patient, normal sample and tumor sample, respectively. (D) The relative expression level of fusion gene IGLV1-51--IGLL5 in HCC samples.

**^#^** Figures are placed together from different regions of the same gel, and separated by a white dotted line.

● represents the verified sample. Data are given as mean ± SEM (n = 3). *p <0.05, **p <0.01, ***p <0.001.

**F****igure S5.** Details of RP11-100N3.2--GNAS after experimental validation of the fusion transcripts by RT-PCR, Sanger sequencing and Quantitative Real-time PCR.

(A-B) The electrophoretic result and sequencing data for RT-PCR product with fusion gene RP11-100N3.2--GNAS. (C) Verified samples for the existence fusion RP11-100N3.2—GNAS. Recur-ratio shows the ratio of the verified sample comparing with the total number in patient, normal sample and tumor sample, respectively. (D) The relative expression level of fusion gene RP11-100N3.2--GNAS in HCC samples.

**^#^** Figures are placed together from different regions of the same gel, and separated by a white dotted line.

● represents the verified sample. Data are given as mean ± SEM (n = 3). *p <0.05, **p <0.01, ***p <0.001.

**F****igure S6.** Details of XXbac-BPG248L24.12--EVA1B after experimental validation of the fusion transcripts by RT-PCR, Sanger sequencing and Quantitative Real-time PCR.

(A-B) The electrophoretic result and sequencing data for RT-PCR product with fusion gene XXbac-BPG248L24.12--EVA1B. (C) Verified samples for the existence fusion XXbac-BPG248L24.12—EVA1B. Recur-ratio shows the ratio of the verified sample comparing with the total number in patient, normal sample and tumor sample, respectively. (D) The relative expression level of fusion gene XXbac-BPG248L24.12--EVA1B in HCC samples.

**^#^** Figures are placed together from different regions of different gels, and separated by a white dotted line.

● represents the verified sample. Data are given as mean ± SEM (n = 3). *p <0.05, **p <0.01, ***p <0.001.

**Figure S7.** Details of IGLV4-69--IGLJ3 after experimental validation of the fusion transcripts by RT-PCR and Sanger sequencing.

(A-B) The electrophoretic result and sequencing data for RT-PCR product with fusion gene IGLV4-69--IGLJ3. (C) Verified samples for the existence fusion IGLV4-69--IGLJ3.

**^#^** Figures are placed together from different regions of the same gel, and separated by a white dotted line.

● represents the verified sample. Recur-ratio shows the ratio of the verified sample comparing with the total number in patient, normal sample and tumor sample, respectively.

**Figure S8.**The fusion events involved in known disease related fusion genes. (A) The breakpoint of known disease fusion C15orf57--CBX3. (B) The breakpoint of AP3D1—SLC6A8.

**Figure S9.**The breakpoint of novel fusion events associated with HCC. (A) The breakpoint of DCUN1D3—GSG1L. (B) The breakpoint of SERPINA5—SERPINA9.

**Figure S10.** The junction reads of two known disease related fusion genes. (A) The junction reads of C15orf57-CBX3. (B) The junction reads of AP3D1-SLC6A8.
